# Traditional Chinese medicine and plant metabolites for rheumatoid arthritis via modulating gut microbiota: a scoping review evaluating the transition from correlation to causality

**DOI:** 10.3389/fphar.2026.1790536

**Published:** 2026-03-13

**Authors:** Xiadong Yang, Rui Niu, Tian Lan, Shouze Ren, Hua Liang, Ying Ma, Chang Liu

**Affiliations:** 1 Graduate School, Heilongjiang University of Chinese Medicine, Harbin, China; 2 Xiyuan Hospital, China Academy of Chinese Medical Sciences, Beijing, China

**Keywords:** rheumatoid arthritis, gut microbiota, Traditional Chinese Medicine, natural products, fecal microbiota transplantation

## Abstract

**Background:**

Rheumatoid arthritis (RA) is an autoimmune disease characterized by chronic synovitis. The “gut-joint axis” proposes gut microbiota and metabolites modulate RA inflammation via mucosal and systemic immune responses. Botanical drugs (Traditional Chinese Medicine, TCM) and plant metabolites offer multi-target potential. However, most studies remain descriptive, demonstrating concurrent microbial shifts but lacking causal designs to verify mechanistic necessity.

**Objectives:**

This scoping review examines TCM and plant metabolite interventions on RA gut microecology (2015–2025), focusing on the “microbiota–metabolite–immune” axis. It aims to classify evidence based on causal design rigor and identify steps to advance research from correlation to causality.

**Methods:**

We searched PubMed, Embase, and Web of Science (2015–2025). Studies reporting RA outcomes and gut microbiota changes following TCM interventions were included. We established a hierarchical classification system based on design rigor: antibiotic depletion (ABX), fecal microbiota transplantation (FMT), metabolite rescue, and blocking. Evidence was stratified: Level A (Closed-loop: ABX + FMT + rescue/blocking), Level A+ (plus *in vitro* blocking), Level B (Partial: ABX/FMT alone), and Level C (Correlational).

**Results:**

Of 25 included studies (24 animal, 1 clinical), only 2 were Level A, 1 Level A+, 3 Level B, and 19 Level C. While TCM improved RA phenotypes and altered microbiota, complete closed-loop verification remains rare. Short-chain fatty acids (SCFAs) show promise but inconsistent trends due to heterogeneity. Bile acids and tryptophan metabolites correlate with reduced inflammation, yet their mechanistic necessity remains largely untested.

**Conclusion:**

Botanical drugs and plant metabolites demonstrate potential in modulating gut microbiota to improve RA. However, definitive causal links remain underexplored. Future research should prioritize “shortest closed-loop” strategies, including targeted quantification, rescue, and necessity validations. Longitudinal designs and systemic immune metrics are essential to transition from correlations to translatable mechanisms.

## 1 Introduction

Rheumatoid arthritis (RA) is a systemic autoimmune disease mainly manifested as erosive damage to joints (Smith and Berman, 2022). It is still a difficult clinical problem to deal with. By 2050, the number of patients with rheumatoid arthritis worldwide is expected to increase to 31.7 million, an amazing 80.2% increase over the current one (Chen S. et al., 2023). This disease is mainly caused by persistent synovitis, which can destroy joint cartilage, erode adjacent bones, and gradually lead to structural damage (Smith and Berman, 2022). Although the clinical manifestations are mainly joint involvement, there may also be extra-joint symptoms, such as vasculitis; in severe cases, the disease will attack important organs - the heart, lungs and kidneys - leading to multi-system damage and high risk of disability (Smolen et al., 2016). Although continuous research has been made, the exact etiology and key pathogenesis are still unclear, but it is generally believed that this is the result of the joint effects of genetic susceptibility, environmental impact and immune system disorders (McInnes and Schett, 2011). Recent evidence also highlights the pathogenic role of neutrophil extracellular traps (NETs) in amplifying synovial inflammation and autoimmunity (Mao et al., 2025). In terms of treatment, non-steroidal anti-inflammatory drugs (NSAIDs) are mainly used to relieve pain and inflammation symptoms, while the control of the disease itself depends on disease-regulating antirheumatic drugs (DMARDs), biological agents and glucocorticoids. (Smolen et al., 2023). However, the safety issues and cumulative side effects of long-term medication will still limit the balance between efficacy and risk (Smolen et al., 2023). So, it is not only advisable but also urgent to find new intervention strategies - those that are low-toxic and synergistic. In recent years, “intestinal-joint axis” has become a key frontier field in the research of rheumatoid arthritis (Zaiss et al., 2021). As the main regulator of the host immune balance, the intestinal microbiota is likely to promote the initiation, amplification and maintenance of inflammation by affecting the integrity of the intestinal mucosal barrier, mucosal immune regulation and systemic inflammatory network (Lin et al., 2023). The key is that, rather than just describing the abundance of microorganisms, what is more important is the functional output of the microbiome - especially the metabolites produced and their signaling effects - is the part that can actually affect the host’s immune regulation (Wang et al., 2023). For example, some metabolites such as short-chain fatty acids (SCFAs) and bile acids will affect the function of immune cells through receptor signaling and epigenetic regulation (Hosseinkhani et al., 2021). When barrier function is impaired or permeability is enhanced, the possibility of these metabolites or microbial-related molecular patterns entering the blood increases, which explains their influence mechanism on peripheral immune response and joint synovial inflammation microenvironment (Abebaw et al., 2025; Jin et al., 2025). Research based on the “microbial-metabolite-immuno-inflammatory” chain is expected to unravel the source of systemic inflammation of rheumatoid arthritis and discover new intervention targets (van der Meulen et al., 2016).

Against this background, traditional Chinese medicine and natural products have occupied a place in the research on the auxiliary treatment and mechanism of rheumatoid arthritis. They have attracted attention for their multi-target regulatory characteristics and potential long-term safety (Long et al., 2021; Xie et al., 2021). Clinical meta-analyses and systematic reviews have further validated the efficacy of TCM in alleviating joint dysfunction and pain in inflammatory arthritis (Li et al., 2023). Previous systematic review and meta-analysis show that traditional Chinese medicine - especially in combination with traditional therapy - may be beneficial in alleviating symptoms and reducing certain inflammatory markers, and the overall safety is not good (Sun et al., 2020; Xing et al., 2020). At the same time, more and more basic studies show that some traditional Chinese medicines or natural products may indirectly affect peripheral immunity and synovial inflammation by regulating the balance of intestinal flora, enhancing the intestinal mucosal barrier, and remodeling the metabolite spectrum (Huang et al., 2025). Indeed, regulating the gut microbiota is increasingly recognized as a novel therapeutic strategy for TCM in RA management (Liang et al., 2023). However, a considerable part of the existing literature is still at the correlation level - that is, “microbiota/metabolite changes and phenotypic improvement occur simultaneously” - lacks closed-loop verification of key mediator molecules and their receptors/pathways. This makes the mechanism inference easy to be over-conjected.

This review systematically summarizes the evidence of the research on the relationship between traditional Chinese medicine/natural product intervention rheumatoid arthritis (RA) and intestinal microecology from 2015 to 2025. Our goal is to answer two key questions: (1) To what extent does the existing evidence support the verifiable causal chain, linking microbiota, metabolites and immune inflammation with the RA phenotype? (2) Which metabolites and pathway modules need to be verified first?To reduce the common error of treating correlation as causation, we propose an evidence grading framework with four levels. Level A requires a complete closed loop, including ABX + FMT + rescue or *in vivo* blocking. Level A+ includes all Level A elements and adds *in vitro* blocking, so the mechanistic link is more specific. Level B provides only partial functional support, such as ABX-only or FMT-only designs. Level C indicates mainly correlational evidence.

Based on this hierarchical structure, we have further built a “causal verification path map”. The figure shows a gradual chain: intervention, flora changes, metabolite transformation, immune/inflammatory pathway readings, which eventually lead to the phytotype of rheumatoid arthritis. We mark each node as verified or unverified. In this way, the intensity of evidence can be compared clearly and transparently, and a verifiable experimental path can be provided for future research.

**FIGURE 1:**
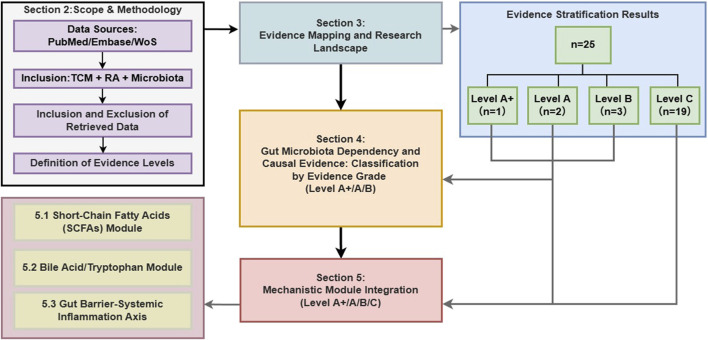
Comprehensive flowchart of literature selection, evidence stratification, and mechanism.

To address the prevalence of correlational data, this review proposes an evidence grading framework grounded in the pharmacological principles of necessity and sufficiency. We aim to distinguish studies that merely show association from those that establish a verifiable causal chain. Specifically, we assess whether the gut microbiota is necessary for the therapeutic effect (verified via antibiotic depletion) and whether the altered microbiota is sufficient to recapitulate the effect (verified via FMT or metabolite rescue). By applying this rigorous filter, we identify which botanical drugs and metabolites have firmly established mechanisms versus those requiring further validation (Figure 1).

## 2 Methods

### 2.1 Search strategy

We have conducted a comprehensive literature search for PubMed, Embase and Web of Science databases. The search time range is limited to 1 January 2015 and 31 December 2025, and the final search will end on 1 January 2026. The retrieval strategy adopts a combination of controlled word list (MeSH/Emtree) and free text words, and is built around three core concepts: (1) rheumatoid arthritis; (2) intestinal flora/microbiome; (3) traditional Chinese medicine/Chinese herbal medicine/prescription/herbal/plant chemical composition. In addition, we have also carried out backreference tracking of the included research and related reviews to retrieve any literature that may be omitted.

The PubMed search strategy was set as follows: (“Arthritis, Rheumatoid” [Mesh] OR rheumatoid arthritis [Title/Abstract] OR RA [Title/Abstract]) AND (“Gastrointestinal Microbiome” [Mesh] OR gut microbiota [Title/Abstract] OR intestinal microbiota [Title/Abstract] OR gut microbiome [Title/Abstract] OR microbiome [Title/Abstract] OR dysbiosis [Title/Abstract]) AND (“Medicine, Chinese Traditional” [Mesh] OR traditional Chinese medicine [Title/Abstract] OR TCM [Title/Abstract] OR Chinese herbal medicine [Title/Abstract] OR herbal medicine [Title/Abstract] OR herb [Title/Abstract] OR formula [Title/Abstract] OR decoction [Title/Abstract] OR phytochemical [Title/Abstract] OR natural product [Title/Abstract]) AND (“2015/01/01” [Date - Publication]: “2025/12/31” [Date - Publication]).

For Embase and Web of Science, we use the specific keywords and grammar rules of their respective platforms to adjust the conceptual framework, and add synonyms such as “intestinal flora” or “fecal microbiota” if necessary to improve sensitivity.

### 2.2 Inclusion and exclusion criteria

#### 2.2.1 Inclusion criteria

1. Subjects/Disease: Rheumatoid arthritis (RA), including both clinical studies (RA patients) and animal model studies (e.g., collagen-induced arthritis [CIA] and adjuvant-induced arthritis [AIA]).
2. Intervention: Interventions centered on Traditional Chinese Medicine (TCM), including formulas/metabolites, single botanical drugs, extracts/active fractions, metabolites, and proprietary Chinese medicines.
3. Control: The study must provide a comparable control group (e.g., model control or healthy control) to evaluate the intervention effect.
4. Microecological Data: Data derived from gut-origin samples (feces/intestinal contents/intestinal mucosa), such as 16S rRNA sequencing, metagenomics, qPCR, culture, or microbial metabolomics.
5. Outcomes: Reports of RA-related clinical outcomes (e.g., disease activity indices, inflammatory markers) or animal RA phenotypic outcomes (e.g., arthritis scores, histology, inflammatory cytokines).
6. Study Type: Original research (clinical or animal/mechanistic studies).

#### 2.2.2 Exclusion criteria

1. Non-RA conditions (e.g., other forms of arthritis or autoimmune diseases where RA is not reported separately).
2. Non-TCM interventions (e.g., pure probiotics/prebiotics unrelated to TCM, or dietary interventions alone).
3. Absence of gut microecology measurements (speculation only, or lack of sequencing/detection).
4. Non-original research (reviews, editorials, or conference abstracts lacking complete data).
5. Data Unavailability: Instances where the full text was unobtainable or core outcome data were missing led to exclusion; however, if only “key grading nodes” were missing, the study was not excluded but was strictly downgraded during the evidence grading process.

### 2.3 Data extraction items

Data mining was a dual-author endeavor. Two authors independently screened titles and evaluated full texts, cross-checking key items collaboratively. When there is a disagreement, we will resolve it through discussion. If we can’t reach an agreement, we will ask the third researcher to arbitrate. We have extracted the following information from each selected study:

Basic Information: Author, year, and study type (clinical or animal), plus the model or study population.

Intervention Details: Intervention category (formula, single botanical drug, extract, or metabolite), the name of the agent, and the administration route.

Microecological Data: Detection method (16S rRNA sequencing, metagenomics, or metabolomics) and the main microecology-related findings.

RA-Related Outcomes: Clinical or animal phenotypes, inflammatory cytokines, and immune-related indices.

Key Information for Evidence Grading: Whether the study used antibiotic depletion or germ-free animals (ABX), fecal microbiota transplantation (FMT), rescue or blocking experiments, and whether engraftment/colonization was verified.

Given that this study is positioned as a scoping review - aimed at describing the structure of evidence and the type of causal design, rather than summarizing the amount of effects or formulating clinical guidelines. As a scoping review aimed at evidence mapping, we systematically extracted data to categorize causal designs. Although risk of bias assessment is not mandatory for scoping reviews, we elected to perform it to provide a comprehensive overview of the current study quality in this field.

### 2.4 Definition of evidence grades

To rigorously assess the pharmacological causality linking botanical interventions, gut microbiota, and RA outcomes, we stratified studies based on the completeness of the experimental chain:

Level A (Closed-Loop Causal Evidence): Studies that establish both the necessity of the microbiota (via antibiotic depletion, ABX) and the sufficiency of specific microbial mediators (via Fecal Microbiota Transplantation [FMT] combined with metabolite rescue or *in vivo* pathway blocking). This design forms a complete closed loop, verifying that the metabolite is the direct effector.

Functional verification can include rescue experiments (for example, giving back key metabolites or other candidate mediators) and/or blocking experiments (for example, blocking key receptors or signaling pathways, or functionally inhibiting key microbial or metabolic pathways). Blocking is separated as *in vivo* and *in vitro*. *In vivo* blocking tests the necessity of a proposed mechanism inside the animal, such as by receptor/pathway antagonists, antibody neutralization, or genetic intervention. This is the key basis for “necessity evidence.” *In vitro* blocking is only used to support mechanistic specificity and consistency. It is not the same as *in vivo* necessity testing.

For practicality of the grading system, we do not use *in vitro* blocking as a strict requirement to upgrade the main level. Instead, we record it as an extra label under Level A studies, as A+ (*in vitro* blocking). We interpret this label strictly as support for mechanistic specificity, not as evidence for *in vivo* necessity, and we discuss it with the same level of caution.

Level B (Partial Functional Evidence): Studies incorporating key manipulation steps—either ABX (indicating microbiota dependence/necessity) or FMT (indicating transferability/sufficiency)—but lacking the complete “depletion-repletion” closed loop to pinpoint specific molecular mediators.

Level C (Correlational Evidence): Studies reporting synchronous changes in microbiota/metabolite profiles and RA phenotypes without functional verification (no ABX, FMT, or rescue). These provide associative data but cannot establish causality.

*A Priori* Judgment Rules (to minimize grading disputes):

1. Studies with ABX + FMT but lacking rescue/*in vivo* blocking are classified as Level B.
2. Only when rescue and/or *in vivo* blocking are incorporated on top of ABX + FMT, forming a testable “Intervention—Microecology—Phenotype” closed loop, is the study classified as Level A.
3. If missing key information prevents the determination of a closed-loop chain, the study is strictly downgraded (typically to Level B; if both ABX and FMT are absent, it falls to Level C).
4. Level A Internal Tagging: The *In vitro* blocking tag is added to Level A studies to indicate further molecular mechanism support (e.g., receptor/pathway verification at the cellular level), forming a dual loop of “*in vivo* function + *in vitro* mechanism.” This tag is for result presentation and interpretative discussion only and does not alter the primary A/B/C classification.

The above contents are shown in Table 1.

**TABLE 1:** Evidence grading criteria for causal inference in the efficacy of TCM and plant metabolites mediated by microbiota.

Grading level	Definition	ABX	FMT	Rescue/*In vivo* blocking	*In vitro* blocking
Level A+	Full Closed-loop (Sufficiency and Necessity)	Yes	Yes	Yes	Yes
Level A	Closed-loop (Sufficiency)	Yes	Yes	Yes	No
Level B	Functional/Dependent	Yes/No*	Yes/No*	No	No
Level C	Correlational	No	No	No	No

Decision Rules and Notes.

Level B Criteria (*): Requires at least one of ABX, or FMT, to be “Yes”. Studies with ABX + FMT, but lacking specific molecular verification (Rescue/*in vivo* Blocking) are classified as Level B.

Level A Criteria: Defined by the presence of Rescue or *In vivo* Blocking. this confirms the causal link in a living system.

Level A + Criteria: Requires the addition of *In vitro* Blocking to provide specific mechanistic support (molecular necessity) on top of the *in vivo* evidence.

### 2.5 Risk of bias assessment

To ensure methodological transparency and assess the quality of the evidence base, we evaluated the risk of bias for the included animal studies (n = 24) using the SYRCLE tool (Hooijmans et al., 2014). Two authors independently evaluated ten domains: sequence generation, baseline characteristics, allocation concealment, random housing, blinding (performance), random outcome assessment, blinding (detection), incomplete outcome data, selective reporting, and other sources of bias. Each domain was scored as “Low risk,” “High risk,” or “Unclear risk.” Disagreements were resolved by consensus or consultation with a third reviewer.

## 3 Evidence map and study overview

### 3.1 General overview of included studies

This review marshaled a total of 25 original studies spanning the decade from 2015 to 2025. This field is almost completely dominated by animal research (24/25, 96.0%), while clinical human research is isolated (1/25, 4.0%).

#### 3.1.1 Intervention types

Classified according to the material form of the intervention, a complete range can be seen, from complex formulas to single metabolites. Specifically, the distribution includes: Formulas/Decoctions/Proprietary Chinese Medicines (13/25, 52.0%), single botanical drug extracts (3/25, 12.0%), polysaccharides (3/25, 12.0%), total flavonoids (2/25, 8.0%), and natural metabolites (4/25, 16.0%).

The formulation, preparation, and taxonomic validation of botanical medicines and their metabolites are presented in Table 2.

**TABLE 2:** Composition, preparation, and taxonomic validation of botanical drugs and plant metabolites.

Intervention	Composition/Origin	Source/Authentication	Preparation	References
Fengshining Decoction (FSN)	Notopterygium incisum K.C.Ting ex H.T.Chang (Apiaceae); Angelica biserrata (R.H.Shan and C.Q.Yuan) C.Q.Yuan and R.H.Shan (Apiaceae); Sinomenium acutum (Thunb.) Rehder and E.H.Wilson (Menispermaceae); Clematis chinensis Osbeck (Ranunculaceae); Curcuma phaeocaulis Valeton (Zingiberaceae); Saposhnikovia divaricata (Turcz.) Schischk. (Apiaceae); Ligusticum striatum DC. (Apiaceae); Ephedra sinica Stapf (Ephedraceae); Cinnamomum cassia (L.) J.Presl (Lauraceae); Sparganium stoloniferum (Graebn.) Buch.-Ham. ex Juz. (Typhaceae); Dracaena cochinchinensis (Lour.) S.C.Chen (Asparagaceae); Corydalis yanhusuo (Y.H.Chou and Chun C.Hsu) W.T.Wang ex Z.Y.Su and C.Y.Wu (Papaveraceae); Achyranthes bidentata Blume (Amaranthaceae); Rehmannia glutinosa (Gaertn.) Libosch. ex Fisch. and C.A.Mey. (Orobanchaceae); Amomum villosum Lour. (Zingiberaceae)	Supplied by Affiliated Hospital of Shanxi University of Traditional Chinese Medicine	Soaked in deionized water 30 min; decocted twice; combined; filtered; concentrated by rotary evaporation to 2 g/mL; stored at 4 °C	Wen et al. (2025)
Er Miao San (EMS)	Atractylodes lancea (Thunb.) DC. (Asteraceae); Phellodendron amurense Rupr. (Rutaceae)	Identified by Dr. Liu SJ (School of Pharmacy, Anhui University of Chinese Medicine); voucher ID: EMS-22-01; deposited in Herbarium of Pharmacy, School of Pharmacy, Anhui University of Chinese Medicine (China)	Dried and crushed; soaked ∼2 h; extracted with 10×/8×/6× water for 1.5 h/1 h/0.5 h; combined filtrates; concentrated at 60 °C; partitioned 5× with equal volumes of petroleum ether and ethyl acetate; concentrated ethyl acetate fraction to required doses (crude drug equivalent)	Xu et al. (2025)
*Acanthopanax senticosus* polysaccharides (ASPS)	Eleutherococcus senticosus (Rupr. and Maxim.) Maxim. (Araliaceae)	Purchased from The First Affiliated Hospital of Soochow University	Water-soluble polysaccharide extracted; purified by anion-exchange and size-exclusion chromatography to obtain ASPS.	Liu et al. (2023)
*Tripterygium hypoglaucum* extract (THH)	Tripterygium hypoglaucum (H.Lév.) Hutch. (Celastraceae)	Provided by Prof. Yanlei Guo; identity authorized by Prof. Yanjiu Liu (Hubei University of Chinese Medicine); voucher specimen No. SM709501666	Roots dried and ground; extracted with water (1:3–5, w/v) at 60 °C for 1–1.5 h, 3 times; filtrated and condensed into paste; re-extracted with 70% ethanol; concentrated into cream (relative density 1.20–1.25); stored at −80 °C	Zheng et al. (2023)
*Aconitum carmichaelii Debx* extract (Fuzi)	Aconitum carmichaelii Debeaux (Ranunculaceae)	Obtained from Sichuan Jiangyou Zhongba Fuzi Keji Fazhan Co., Ltd. (Jiangyou, China)	Immersed in 10× water for 30 min; boiled in water for 5 h (detoxification as described)	Liu et al. (2023)
Jingfang Granules (JFG)	Schizonepeta tenuifolia (Benth.) Briq. (Lamiaceae); Saposhnikovia divaricata (Turcz.) Schischk. (Apiaceae); Notopterygium incisum K.C.Ting ex H.T.Chang (Apiaceae); Heracleum hemsleyanum Diels (Apiaceae); Bupleurum chinense DC. (Apiaceae); Peucedanum praeruptorum Dunn (Apiaceae); Ligusticum striatum DC. (Apiaceae); Citrus aurantium L. (Rutaceae); Wolfiporia cocos (F.A.Wolf) Ryvarden and Gilb. (Polyporaceae) [Fungus]; Platycodon grandiflorus (Jacq.) A.DC. (Campanulaceae); Glycyrrhiza uralensis Fisch. ex DC. (Fabaceae)	Supplied by Shandong New Time Pharmaceutical Co., LTD. (Linyi, Shandong, China); batch No. Z37020357	Commercial granules; no additional preparation reported	Wang et al. (2025b)
Wu-tou Decoction (WTD)	Aconitum carmichaelii Debeaux (Ranunculaceae); Ephedra sinica Stapf (Ephedraceae); Glycyrrhiza uralensis Fisch. ex DC. (Fabaceae); Astragalus membranaceus (Fisch.) Bunge (Fabaceae); Paeonia lactiflora Pall. (Paeoniaceae)	Purchased from Ji Lin Pharmacy; identified by Prof. Shumin Wang (Changchun University of Chinese Medicine); voucher specimens stored in Changchun Institute of Applied Chemistry, CAS (No. 20180603)	Total 1400 g crude herbs (ratio 2:3:3:3:3); immersed in 14 L water 1 h; reflux extracted 1.5 h; second reflux extraction with 11.2 L water 1.5 h; combined extracts; concentrated to 1.5 g/mL	Cheng et al. (2022)
Wu-tou Decoction (WTD); fractions (SM vs. PS)	Aconitum carmichaelii Debeaux (Ranunculaceae); Ephedra sinica Stapf (Ephedraceae); Glycyrrhiza uralensis Fisch. ex DC. (Fabaceae); Astragalus membranaceus (Fisch.) Bunge (Fabaceae); Paeonia lactiflora Pall. (Paeoniaceae)	Purchased from Changchun University of Chinese Medicine, No. 1035 Boshuo Rd	1400 g crude herbs (ZCW 200 g; MH 300 g; GC 300 g; BS 300 g; HQ 300 g); immersed 1 h; reflux extracted twice (14 L and 11.2 L water, 1.5 h each); combined extracts; half concentrated to 1.5 g/mL (stored); remaining half concentrated to 0.5 g/mL then ethanol added to 70% for overnight precipitation; centrifuged to separate SM and PS; SM concentrated to 1.5 g/mL (crude drug equivalent); PS dissolved in water and concentrated to 1.5 g/mL (crude drug equivalent)	Yang et al. (2024)
Yaobitong capsule (YBTC)	Panax notoginseng (Burkill) F.H.Chen (Araliaceae); Cibotium barometz (L.) J.Sm. (Cibotiaceae); Corydalis yanhusuo W.T.Wang (Papaveraceae); Cynanchum otophyllum C.K.Schneid. (Apocynaceae); Angelica biserrata (R.H.Shan and C.Q.Yuan) C.Q.Yuan and R.H.Shan (Apiaceae); Achyranthes bidentata Blume (Amaranthaceae); Rheum palmatum L. (Polygonaceae); Ligusticum striatum DC. (Apiaceae)	Supplied by Jiangsu Kanion Pharmaceutical Co., Ltd (Jiangsu, China); Lot No. 1802112876	Commercial capsule; no additional preparation reported	Shi et al. (2022)
Wantong Jingu Tablet (WJT)	Aconitum carmichaelii Debeaux (Ranunculaceae); Aconitum kusnezoffii Rchb. (Ranunculaceae); Strychnos nux-vomica L. (Loganiaceae); Epimedium brevicornu Maxim. (Berberidaceae); Achyranthes bidentata Blume (Amaranthaceae); Notopterygium incisum K.C.Ting ex H.T.Chang (Apiaceae); Dryopteris crassirhizoma Nakai (Dryopteridaceae); Phellodendron amurense Rupr. (Rutaceae); Dipsacus asper Wall. ex C.B.Clarke (Caprifoliaceae); Prunus mume (Siebold) Siebold and Zucc. (Rosaceae); Asarum sieboldii Miq. (Aristolochiaceae); Ephedra sinica Stapf (Ephedraceae); Cinnamomum cassia (L.) J.Presl (Lauraceae); Carthamus tinctorius L. (Asteraceae); Eleutherococcus senticosus (Rupr. and Maxim.) Maxim. (Araliaceae); *Lonicera japonica* Thunb. (Caprifoliaceae); Taxillus sutchuenensis (Lecomte) Danser (Loranthaceae); Glycyrrhiza uralensis Fisch. ex DC. (Fabaceae); Davallia mariesii T.Moore ex Baker (Davalliaceae); Illicium difengpi B.N.Chang (Schisandraceae); Commiphora myrrha (Nees) Engl. (Burseraceae); Panax ginseng C.A.Mey. (Araliaceae)	Obtained from Jilin Wantong Pharmacy Group Company (Tonghua, China)	Commercial tablets; no additional preparation reported	Li et al. (2022c)
Siweixizangmaoru decoction (SXD)	Rhamnella gilgitica Mansf. and Melch. (Rhamnaceae); Berberis thunbergii DC. (Berberidaceae); Gentiana macrophylla Pall. (Gentianaceae); Terminalia chebula Retz. (Combretaceae)	Purchased from Manchester Biotechnology Co., Ltd. (Chengdu, China)	Extracted RG, GM, BT, and TC (12:10:6.5:3, w/w) in distilled water (1:15, w/v) at 100 °C twice (1.5 h each); filtration; freeze-drying; stored at −20 °C	Sun et al. (2025)
*Flemingia philippinensis* total flavonoids (FPTF)	Flemingia philippinensis Merr. and Rolfe (Fabaceae)	Purchased from Lingnan Traditional Chinese Medicine Co., LTD. (Batch No. 2022001); authenticated by Prof. Zhang Danyan (Chinese Medicine College of Guangzhou University of Chinese Medicine)	Pulverized; soaked in 10 %× 70% ethanol for 24 h; extracted twice (2 h each); combined filtrates concentrated under reduced pressure until ethanol removed; stood 2 h then suction filtration; precipitate washed 3× with deionized water; purified using AB-8 macroporous resin; filtered, concentrated, and freeze-dried; stored at −20 °C; stock solution prepared in methanol (5 mg/mL) and centrifuged (14,000 rpm, 10 min)	Qiu et al. (2025)
Ershiwuwei Lvxue Pill (ELP)	Justicia adhatoda L. (Acanthaceae); Syzygium aromaticum (L.) Merr. and L.M.Perry (Myrtaceae); Syzygium cumini (L.) Skeels (Myrtaceae); Wulfenia carinthiaca Jacq. (Plantaginaceae); Dracocephalum moldavica L. (Lamiaceae); Althaea officinalis L. (Malvaceae); Dalbergia odorifera T.C.Chen (Fabaceae); Sesamum indicum L. (Pedaliaceae); Trema tomentosa (Roxb.) H.Hara (Cannabaceae); Saussurea costus (Falc.) Lipsch. (Asteraceae); Eriogonum inflatum Torr. and Frém. (Polygonaceae); Terminalia bellirica (Gaertn.) Roxb. (Combretaceae); Berberis vulgaris L. (Berberidaceae); Prosopis cineraria (L.) Druce (Fabaceae); Phyllanthus emblica L. (Phyllanthaceae); Boswellia serrata Roxb. ex Colebr. (Burseraceae); Morus alba L. (Moraceae); Melilotus officinalis (L.) Pall. (Fabaceae); Santalum album L. (Santalaceae); Rheum palmatum L. (Polygonaceae); Bassia scoparia (L.) A.J.Scott (Amaranthaceae); Phyllanthus niruri L. (Phyllanthaceae)	Purchased from Tibet Ganlu Tibetan Medicine Co., Ltd. (Tibet, China); SFDA approval No. Z54020070; #19,104	Commercial pills; no additional preparation reported	Li et al. (2022a)
Dianbaizhu (*Gaultheria leucocarpa* var. *yunnanensis (Franch.) T. Z. Hsu and R. C. Fang*) polysaccharides (DBZP)	Gaultheria leucocarpa var. yunnanensis (Franch.) T.Z.Hsu and R.C.Fang (Ericaceae)	Purchased from Chuxiong (Yunnan, China); authenticated by Prof. Shengli Wei (School of Chinese Materia Medica, Beijing University of Chinese Medicine)	Aerial parts refluxed with 95% ethanol for 4 h; residue dried then extracted with water (1:20, w/v) at 100 °C for 3 h, repeated once; filtrates concentrated under reduced pressure at 48 °C; ethanol added to 80% for precipitation; centrifuged (4000 rpm, 15 min); precipitation step repeated three times; precipitate washed with anhydrous ethanol/acetone/anhydrous ether; solvent evaporated; freeze-dried to obtain DBZP.	Dong et al. (2024)
Berberine (BBR)	The intervention is a plant-derived metabolite. Berberine is an isoquinoline alkaloid extracted from medicinal plants such as Coptis chinensis Franch. (Ranunculaceae) and Berberis spp. (Berberidaceae)	Purchased from Zelang Pharmaceutical Technology Co., Ltd. (Nanjing, China)	Commercial metabolite; no additional preparation reported	Li et al. (2024)
Twenty-five Wei’er tea pill (TFP)	Twenty-five Wei’er tea pills (TFP), made of 25 Tibetan natural drugs (mostly botanical drugs), such as: Senegalia catechu (L.f.) P.J.H.Hurter and Mabb. (Fabaceae); Terminalia chebula Retz. (Combretaceae); Terminalia bellirica (Gaertn.) Roxb. (Combretaceae); Phyllanthus emblica L. (Phyllanthaceae); Hymenidium hookeri (C.B.Clarke) Pimenov and Kljuykov (Apiaceae); Polygonatum sibiricum Redouté (Asparagaceae); Asparagus cochinchinensis (Lour.) Merr. (Asparagaceae); Oxybaphus himalaicus Edgew. (Nyctaginaceae); Tribulus terrestris L. (Zygophyllaceae); Boswellia sacra Flück. (Burseraceae); Senna tora (L.) Roxb. (Fabaceae); Abelmoschus manihot (L.) Medik. (Malvaceae); Tinospora sinensis (Lour.) Merr. (Menispermaceae); Piper longum L. (Piperaceae); Acorus calamus L. (Acoraceae); Dolomiaea costus (Falc.) Kasana and A.K.Pandey (Asteraceae); Oxytropis kansuensis Bunge (Fabaceae); Rosa sweginzowii Hemsl. and E.H.Wilson (Rosaceae); Gentiana macrophylla Pall. (Gentianaceae); Aconitum pendulum N.Busch (Ranunculaceae)	Purchased from Tibet Ganlu Tibetan Medicine Co., Ltd. (Lhasa, China); batch No. 20200502	Commercial pills; no additional preparation reported	Li et al. (2022b)
Tinosporine (TIN)	Tinospora sinensis (Lour.) Merr. (Menispermaceae)	Purchased from Chengdu Alfa Biological Technology Co., Ltd. (Chengdu, China); product No. AFBG1318	Commercial metabolite; no additional preparation reported	Liu et al. (2025a)
Cannabidiol (CBD)	Cannabis sativa L. (Cannabaceae)	Derived from Cannabis sativa L. (Cannabaceae).; the specific supplier has not been specified	The chemical structure is well-defined (PubChem CID: 644019)	Geng et al. (2025)
Ephedra sinica polysaccharide (ESP)	Ephedra sinica Stapf (Ephedraceae)	Purchased from Department of Basic Medical Sciences, Shanxi University of Chinese Medicine, Taiyuan, China; material noted as Herba Ephedra sinica Stapf (401003136P)	Decoction; filtrate concentrated to 0.5–1 g/mL; 95% ethanol added (1:9, v/v) for precipitation 30 min; centrifuged (3500 rpm, 4 °C, 10 min), repeated 2–3 times; filtered; rotary evaporation to yield ESP powder	Ma et al. (2024)
Danggui Sini Decoction (DSD)	Angelica sinensis (Oliv.) Diels (Apiaceae); Cinnamomum cassia (L.) J.Presl (Lauraceae); Paeonia lactiflora Pall. (Paeoniaceae); Tetrapanax papyrifer (Hook.) K.Koch (Araliaceae); Asarum sieboldii Miq. (Aristolochiaceae); Glycyrrhiza uralensis Fisch. ex DC. (Fabaceae); Ziziphus jujuba Mill. (Rhamnaceae)	Purchased from Nanning Shengyuantang Chinese Herbal Medicine Co., Ltd. (Nanning, China); authenticated by Assoc. Prof. Changming Mo (Guangxi Botanical Garden of Medicinal Plants)	Cleaned/dried/cut; 100 g extracted twice with distilled water: 600 mL (100 °C, 2 h) and 400 mL (100 °C, 1.5 h); filtration; vacuum evaporation to 1.0 g/mL stock; stored at −20 °C	He et al. (2023)
Columbianadin (CBN)	Angelica biserrata (R.H.Shan and C.Q.Yuan) C.Q.Yuan and R.H.Shan (Apiaceae)	APR purchased from Anguo City, Hebei, China; columbianadin isolated in laboratory (Tianjin, China)	Isolated from APR (details not further reported)	Chen et al. (2023b)
*Atractylodes koreana* (Nakai) Kitam extract	Atractylodes koreana (Nakai) Kitam. (Asteraceae)	Purchased from Benxi Traditional Chinese Medicine Market; authenticated by Prof. Li Xiangri (Liaoning University of Traditional Chinese Medicine); voucher specimen No. 200918061201 deposited in the herbarium of Liaoning University of Traditional Chinese Medicine; identity cross-checked with The Plant List (http://www.theplantlist.org)	Sliced, dried, and ground (through 180-mesh sieve); prepared suspensions at 0.05/0.2/0.45 g/mL; methotrexate tablets (batch No. h31020644) ground and prepared as 0.05 mg/mL solution; shaken before use	Pang et al. (2021)
Clematis total saponins (CTSs)	Clematis mandshurica Rupr. (Ranunculaceae)	Department of Chinese Medicines Analysis	Prepared and isolated from Clematis mandshurica Rupr	Guo et al. (2019)
Huayu-Qiangshen-Tongbi formul (HQT)	*Salvia miltiorrhiza* Bunge (Lamiaceae); *Dioscorea nipponica* Makino (Dioscoreaceae); *Astragalus membranaceus* (Fabaceae); *Paeonia lactiflora* Pall. (Paeoniaceae); *Saussurea involucrata* (Kar. et Kir.) Sch.-Bip (Asteraceae); *Eucommia ulmoides* Oliver (Eucommiaceae); *Davallia mariesii* Moore ex Bak (Davalliaceae); *Dipsacus asperoides* C. Y. Cheng et T. M. Ai (Caprifoliaceae); *Rehmannia glutinosa* (Gaertn.) DC. (Orobanchaceae); *Glycyrrhiza uralensis* Fisch. ex DC. (Fabaceae)	The Second Affiliated Hospital of Guangzhou University of Chinese Medicine	The decoction was prepared by soaking herbs for 30 min, boiling twice (1200 mL cold water for 40 min; 800 mL hot water for 30 min), filtering twice each time, then combining the filtrates	Mei et al. (2021)
Duzheng Pian (DZGP)	*Eucommia ulmoides* Oliver (Eucommiaceae)	Obtained from Hubei Province Pharmaceutical Preparation; No. Z20210135; originating from the Tujia ethnic group in Enshi	Commercial pills; no additional preparation reported	Zhao et al. (2024)

#### 3.1.2 Microbiome and metabolic detection technologies

Regarding microbiome detection, this field mainly relies on 16S rRNA/rDNA sequencing (23/25, 92.0%), while macrogenome spending is still relatively rare (2/25, 8.0%; mainly appears in ESP research and HQT clinical trials). In terms of metabolic/functional analysis, most studies (23/25, 92.0%) will measure metabolites or functional pathways. However, the degree of measurement varies greatly, including: SCFAs (such as acetic acid, propionic acid, butyric acid determined by GC-MS); bile acid spectrum (such as TCA, THDCA or various free/bined forms, through LC-MS/MS); non-targeted metabolomics (UHPLC-Q-TOF/Orbitrap); and macrogenome functional pathways (such as MetaCyc pathways in HQT research).

### 3.2 Evidence stratification results

The layered results are very interesting. Among the 25 studies included, the hierarchical distribution is as follows: A-level (2 studies, accounting for 8.0%), A+ (1 study, accounting for 4.0%), B-level (3 studies, 12.0%), C-level (19 studies, accounting for 76.0%).

Level A+ (n = 1) and Level A (n = 2): closed-loop causal evidence. These studies strictly combine microbiota consumption (ABX) and fecal bacteria transplantation (FMT), and further integrate rescue or *in vivo* blocking experiments to form at least one closed-loop structure of “intervention-microbiota-phenotype”. This structure supports the causal inference of “microbial dependence” of the therapeutic effect of traditional Chinese medicine. Interestingly, one of the studies went further, including *in vitro* blocking experiments, so it was rated A+.

Level B (n = 3): Some functional evidence. These studies contain at least one key step - either antibiotics (ABX) or fecal bacteria transplantation (FMT) - suggesting that the efficacy may depend on the microbiota or be transferable. However, due to the lack of closed-loop verification through rescue/blocking, this limits the intensity of causal inference.

Level C (n = 19): correlation evidence. In these studies, the report shows that changes in the microbiota/metabolite spectrum after traditional Chinese medicine intervention are parallel to the improvement of rheumatoid arthritis phenotype. Due to the lack of ABX, FMT or rescue/blocking design, these findings only support correlation. However, this level provides fertile soil for generating assumptions about candidate microorganisms, metabolites and future verification paths.

On the whole, the structure of evidence presents a pattern: “Correlation evidence dominates, while closed-loop causal evidence is still scarce.” This highlights that the field is currently in a transitional stage, from exploratory genomic research to falsible causal mechanism research.

**FIGURE 2:**
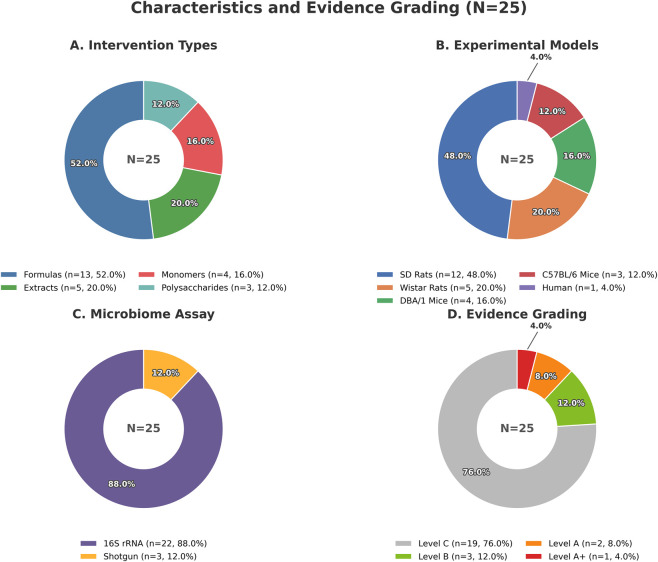
Characteristic overview and evidence stratification included in the study (N = 25). **(A)** Intervention types. **(B)** Experimental models. **(C)** Microbiome assay. **(D)** Evidence grading.

The above contents are shown in Figure 2.

### 3.3 Evidence grade backfill checklist

The content for this paragraph is provided in Table 3.

**TABLE 3:** The characteristics of the research include: intervention type, animal model, changes in microbiota and causal verification experiments.

Intervention	Intervention type	Experimental model	Treatment	Microbiome assay	Metabolomics sample source	ABX	FMT	Rescue	References
Level A+ (n = 1)									
Fengshining Decoction (FSN)	Formula	C57BL/6J; female; CIA	FSN; 20/40/80 g/kg/day; oral gavage; once daily; 21 days; start NR	16S rRNA gene amplicon sequencing	Serum; intestinal contents; feces	Yes	Yes	Yes (butyrate)	Wen et al. (2025)
Level A (n = 2)									
Er Miao San (EMS)	Formula	DBA/1; male; CIA	EMS; 2 g/kg/day; oral gavage; once daily; 28 days; starting day 28 after booster	16S rRNA gene amplicon sequencing	Cecal contents	Yes	Yes	Yes (butyrate)	Xu et al. (2025)
*Acanthopanax senticosus* polysaccharides (ASPS)	Polysaccharide metabolite	DBA/1; male; CIA	Not reported	16S rRNA gene amplicon sequencing	Serum; feces	Yes	Yes	Yes (GGC)	Liu et al. (2023)
Level B (n = 3)									
*Tripterygium hypoglaucum* extract (THH)	Single botanical drug extract from Tripterygium hypoglaucu	C57BL/6; sex NR; CFA	THH extract; 250 mg/kg/day; oral gavage; once daily; 35 days; start NR	16S rRNA gene amplicon sequencing	Feces	Yes	No	No	Zheng et al. (2023)
*Aconitum carmichaelii Debx* extract (Fuzi)	Single botanical drug extract from Aconitum carmichaelii Debx	Wistar; male; CIA + cold exposure	Fuzi aqueous extract; start day 16; oral gavage; once daily; 16 days; day 16–end	16S rRNA gene amplicon sequencing	Serum; feces	No	Yes	No	Liu et al. (2023)
Jingfang Granules (JFG)	proprietary Chinese medicine	Sprague–Dawley; male; CFA	JFG; 0.5/1.0/2.0 g/kg; oral gavage; once daily; 21 days; start NR	16S rRNA gene amplicon sequencing	Serum; feces	Yes	No	No	Wang et al. (2025b)
Level C (n = 19)									
Wu-tou Decoction (WTD)	Formula	Sprague–Dawley; male; AIA	WTD aqueous extract; 9.8 g/kg; oral gavage; once daily; 1 month; start NR	16S rRNA gene amplicon sequencing	Serum; feces	No	No	No	Cheng et al. (2022)
Wu-tou Decoction (WTD); fractions (SM vs. PS)	Formula	Sprague–Dawley; male; AIA (CFA)	WTD/SM/PS; 9.8 g crude drug/kg/day; oral gavage; once daily; 30 days; start NR	16S rRNA gene amplicon sequencing	Feces	No	No	No	Yang et al. (2024)
Yaobitong capsule (YBTC)	proprietary Chinese medicine	Sprague–Dawley; male; AIA (CFA)	YBTC; 2.0 g/kg; oral gavage; once daily; 27 days (day 1–27 reported); start day 0 (from immunization)	16S rRNA gene amplicon sequencing	Feces	No	No	No	Shi et al. (2022)
Wantong Jingu Tablet (WJT)	proprietary Chinese medicine	Sprague–Dawley; female; CIA	WJT; 150/300/600 mg/kg; oral gavage; once daily; 28 days; starting day 1	16S rRNA gene amplicon sequencing	Serum	No	No	No	Li et al. (2022c)
Siweixizangmaoru decoction (SXD)	proprietary Chinese medicine	Sprague–Dawley; male; CIA	SXD; 1.1475/2.835/5.67 g/kg; oral gavage; once daily; 4 weeks; starting day 21	16S rRNA gene amplicon sequencing	Colonic contents	No	No	No	Sun et al. (2025)
*Flemingia philippinensis* total flavonoids (FPTF)	Extract (total flavonoids)	Wistar; female; CIA	FPTF; 18/36/72 mg/kg; oral gavage; once daily; 28 days; starting day 14 (day 14–42)	16S rRNA gene amplicon sequencing	Serum; feces	No	No	No	Qiu et al. (2025)
Ershiwuwei Lvxue Pill (ELP)	proprietary Chinese medicine	Wistar; sex NR; CIA	ELP; 115/230/460 mg/kg; intragastric gavage; once daily; 28 days; start NR	16S rRNA gene amplicon sequencing	Serum	No	No	No	Li et al. (2022a)
Dianbaizhu (*Gaultheria leucocarpa* var. *yunnanensis (Franch.) T. Z. Hsu and R. C. Fang*) polysaccharides (DBZP)	Polysaccharide metabolite	DBA/1J; male; CIA	DBZP; 0.44 or 1.76 g/kg/day; oral gavage; once daily; day 29–57; starting day 29	16S rRNA gene amplicon sequencing	Feces; urine	No	No	No	Dong et al. (2024)
Berberine (BBR)	alkaloid	Sprague–Dawley; male; CIA	BBR; 200 mg/kg/day; oral administration; once daily; 7 weeks; start NR	16S rRNA gene amplicon sequencing	Serum	No	No	No	Li et al. (2024)
Twenty-five Wei’er tea pill (TFP)	proprietary Chinese medicine	Sprague–Dawley; male; CIA	TFP; 150 or 450 mg/kg/day; oral gavage; once daily; 4 weeks; start NR	16S rRNA gene amplicon sequencing	Serum	No	No	No	Li et al. (2022b)
Tinosporine (TIN)	Isolated metabolite (metabolite)	Wistar; male; CIA	TIN; 20/40/80 mg/kg; oral gavage; once daily; 22 days; starting day 8 (day 8–30)	16S rRNA gene amplicon sequencing	Feces	No	No	No	Liu et al. (2025a)
Cannabidiol (CBD)	Isolated metabolite (metabolite)	Sprague–Dawley; male; CIA	CBD; 5 or 10 mg/kg; intragastric gavage; once daily; 21 days; starting day 14	Shotgun metagenomic sequencing (MGS)	Serum	No	No	No	Geng et al. (2025)
Ephedra sinica polysaccharide (ESP)	Single metabolite from Ephedra sinica Stapf	C57BL/6J; sex NR; CIA	ESP; 200 or 400 mg/kg; oral administration; once daily; 21 days; start NR	Shotgun metagenomic sequencing (MGS)	Serum; feces	No	No	No	Ma et al. (2024)
Danggui Sini Decoction (DSD)	Formula	Sprague–Dawley; male; CIA + cold stimulation	DSD; 10.8, 5.4, or 2.7 g/kg/day; oral gavage; once daily; 28 days; starting after CIA model establishment	16S rRNA gene amplicon sequencing	Serum; feces	No	No	No	He et al. (2023)
Columbianadin (CBN)	Coumarin (isolated from Angelicae pubescentis radix; purity >98%)	DBA/1; male; CIA	CBN:20 mg/kg/day; oral gavage; once daily; 21 days; starting day 22 after booster	16S rRNA gene amplicon sequencing	Urine; serum	No	No	No	Chen et al. (2023b)
*Atractylodes koreana* (Nakai) Kitam extract	Single botanical drug extract from Atractylodes koreana (Nakai) Kitam	Sprague–Dawley; male; AA (FCA)	A. koreana aqueous extract; low/medium/high (0.05/0.2/0.45 g/mL); oral gavage 10 mL/kg; once daily; 14 days; start NR	16S rRNA gene amplicon sequencing	Colonic contents; plasma	No	No	No	Pang et al. (2021)
Clematis total saponins (CTSs)	Total saponins (extracted from Clematis spp.)	Wistar; male; CIA	CTSs; 50 or 100 mg/kg; oral administration; once daily; 28 days; start NR	16S rRNA gene amplicon sequencing	Colonic contents	No	No	No	Guo et al. (2019)
Huayu-Qiangshen-Tongbi formul + methotrexat HQT + MTX (vs. LEF + MTX)	Formula	Human; RA patients (2010 ACR/EULAR criteria); sex NR; longitudinal cohort; n = 22 RA (13 HQT + MTX, 9 LEF + MTX) + 22 HC	HQT; once every 2 days; oral; 6 months; combined with MTX 10–15 mg/week; start baseline after enrollment	Shotgun metagenomic sequencing (MGS)	Feces	No	No	No	Mei et al. (2021)
Duzheng Pian (DZGP)	proprietary Chinese medicine	Sprague–Dawley (SD) rats; male; CIA	DZGP; 227 or 908 mg/kg; oral administration; once daily; 28 days; start after arthritis onset	16S rRNA gene amplicon sequencing	Serum	No	No	No	Zhao et al. (2024)

### 3.4 Risk of bias assessment results

**FIGURE 3:**
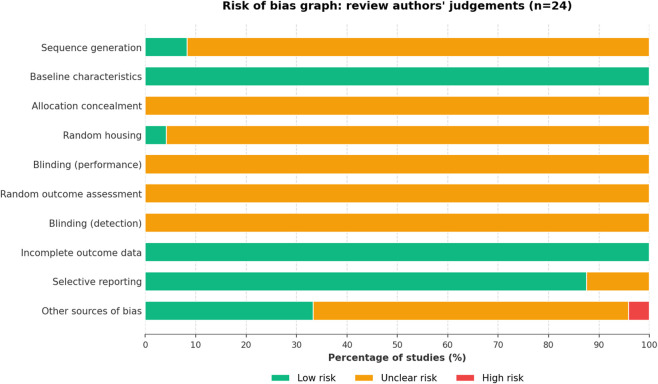
Risk of bias assessment summary. The stacked bar chart illustrates the proportion of studies classified as low risk (green), unclear risk (yellow), and high risk (red) across the ten domains of the SYRCLE’s risk of bias tool (n = 24).

The risk of bias assessment for the 24 included animal studies is summarized in Figure 3. Overall, the methodological quality was variable across the ten SYRCLE domains. Selection Bias: While baseline characteristics were generally well-balanced (24/24 Low risk), details regarding sequence generation (22/24 Unclear) and allocation concealment (24/24 Unclear) were largely missing, leading to a predominance of “Unclear risk” in the selection process. Performance and Detection Bias: Measures to reduce bias during the experiment were poorly reported. Information on random housing, blinding (performance), random outcome assessment, and blinding (detection) was rarely provided, with the majority of studies rated as “Unclear risk” in these four domains. Attrition and Reporting Bias: Most studies adequately addressed incomplete outcome data (24/24 Low risk). Regarding selective reporting, the majority were evaluated as low risk (21/24); however, the lack of pre-registered protocols limits definitive verification. Other Bias: Finally, for other sources of bias, a minority of studies (1/24) were flagged for high risk, while the remainder were classified as either unclear or low risk.

The above contents are shown in Figure 3.

## 4 Microbiome dependence and causal evidence: graded verification system (A-level +/A/B)

### 4.1 Level A + studies: closed-loop evidence + mechanistic pathway intervention (ABX → FMT → rescue/in vivo blocking + *in vitro* blocking)

#### 4.1.1 Fengshining decoction (FSN)

ABX (Low-Biomass Background/Modeling Foundation): This study established a strict low-microbial baseline by taking four antibiotic mixtures continuously for 14 days before modeling, and confirmed a significant decrease in the diversity and number of microorganisms through 16S sequencing verification (Wen et al., 2025). This step lays a solid foundation for the subsequent recovery of FMT and metabolites (Wen et al., 2025).

FMT (Transferability Test): After antibiotic treatment, three groups were established: FSN donor fecal bacteria transplantation group, model donor fecal bacteria transplantation group and antibiotic control group, and FSN intervention was carried out at the same time (daily gastric irrigation for 21 days). The FSN-FMT group had significant improvements in arthritis indicators, inflammatory factors and pathology, and the structure of the intestinal flora gradually approached the FSN donor; in contrast, the improvement of the model donor FMT group and the antibiotic control group was limited (Wen et al., 2025). This design supports the statement that ‘FSN-induced microbial state has a transferable therapeutic effect.'

Rescue (Sufficiency Support for Key Mediators): Adding butyrate supplement group (adding sodium butyrate to drinking water, 200 mmol/L, 21 days) can restore the level of butyric acid in the body and relieve inflammation, and the content of butyric acid is negatively correlated with inflammation markers (Wen et al., 2025). This roughly outlines a clear candidate path: “microbiota - butyric acid - inflammation/joint damage”.

*In Vitro* Blocking: This study also used TSA (HDAC1/2 inhibitor, 100 nM) and PDTC (NF-κB inhibitor, 100 nM) to intervene in cell pathways. Both inhibitors partially suppressed p65 and HDAC-related inflammatory expression in synoviocytes. Western blot analysis revealed that FSN, TSA, and PDTC all inhibited the protein expression of p65, P-p65, HDAC1, and HDAC2; flow cytometry showed that both FSN-containing serum and inhibitors increased the apoptosis rate of synovial fibroblasts; and phalloidin staining demonstrated that while TNF-α stimulation enhanced F-actin fluorescence and promoted pseudopodia formation, FSN and the inhibitors mitigated these abnormalities (Wen et al., 2025). These results reinforce the evidence for the HDAC/NF-κB axis in FSN’s anti-inflammatory effect from the perspective of “pathway intervention consistency/mechanistic module convergence.”

Limitations: Whether butyrate supplementation is truly equivalent to the holistic effect of FSN lacks a strict dose-response control. Furthermore, the *in vitro* blocking evidence primarily strengthens mechanistic specificity rather than strictly proving *in vivo* therapeutic necessity. Future studies should introduce *in vivo* pathway intervention within the ABX/FMT causal framework to solidify these conclusions.

### 4.2 Level a studies: causal chain closed-loop evidence (ABX → FMT → rescue/in vivo blocking)

#### 4.2.1 Er miao san (EMS)

ABX (Microbiota Dependence/Necessity Signal): The study utilized an antibiotic cocktail (ABX) to deplete or significantly perturb the gut microbiota of recipient mice, testing whether EMS efficacy relies on microbial presence (Xu et al., 2025). While data showed that ABX altered the microbial structure, the depletion efficiency was not verified by absolute quantitative metrics like 16S copy number qPCR or colony counting, leaving the intensity of the low-biomass background somewhat uncertain (Xu et al., 2025).

FMT (Transferability Test): Following ABX treatment, two types of donor FMTs were administered: from the EMS treatment group and the CIA model group. PCoA analysis indicated that the recipient microbiota structure shifted toward the donor profile, supporting the notion that “the EMS-induced microbial state can be transplanted and partially reconstructed in the recipient.” (Xu et al., 2025).

Rescue (Metabolite Supplementation to Support Sufficiency): Sodium butyrate (SB) supplementation (200 mg/kg, gavage, 28 days) was found to mimic the anti-inflammatory and joint-improving effects of EMS to a certain extent. Metabolically, the authors emphasized that EMS primarily restored cecal butyrate levels, with limited impact on other SCFAs (Xu et al., 2025). Overall, the data support “butyrate as one of the candidate key mediators,” rather than the sole mediator.

Limitations: The ABX depletion lacked absolute quantitative verification. Moreover, the absence of an effect intensity comparison between SB supplementation, FMT, and the original EMS formula within the same framework limits the quantitative persuasiveness of the closed-loop evidence.

#### 4.2.2 Acanthopanax senticosus polysaccharides (ASPS)

ABX (Microbiota Dependence/Necessity Signal): A quadruple antibiotic cocktail (ampicillin, vancomycin, neomycin, metronidazole; via drinking water for 14 days) was used to significantly deplete the gut microbiota, confirmed by a marked drop in 16S sequencing abundance/diversity (Liu et al., 2023). Against this low-biomass backdrop, the improvement in arthritis phenotypes by ASPS was notably blunted, and serum γ-glutamylcysteine (GGC) levels fell, suggesting that both ASPS efficacy and GGC generation are microbiota-dependent (Liu et al., 2023).

FMT (Transferability Test): Under the ABX background, FMT was performed using feces from the ASPS-treated group, with CIA model donor FMT as a control (daily gavage, 14 days) (Liu et al., 2023). Results showed significant amelioration of arthritis symptoms in ASPS-FMT recipients, whereas model-FMT or ABX controls showed limited improvement, suggesting that “the ASPS-associated microbial configuration possesses a transferable therapeutic effect.”

Rescue (Sufficiency Verification of Key Mediator via Exogenous Supplementation): To test if GGC is the key mediator, the authors exogenously supplemented GGC in CIA model mice (100 mg/kg/day, oral; starting from primary immunization). GGC treatment replicated the joint-protective effects of ASPS: inhibition of arthritis scores and paw thickness, effective suppression of bone erosion activity, and alleviation of cartilage damage and synovial inflammation. Concurrently, serum IL-1β levels dropped significantly, and caspase-1 and IL-1β expression decreased by over 80% (Liu et al., 2023). Combined with the finding that “ABX reduces GGC and weakens ASPS efficacy,” this rescue experiment provides strong sufficiency support for the candidate mechanism chain: “Microbiota Regulation → GGC Elevation → NLRP3 Inflammasome Suppression → Phenotype Improvement.”

Limitations: Although GGC supplementation replicated key phenotypes *in vivo*, the study did not include *in vivo* blocking verification for GGC generation/transport or the NLRP3 module. Thus, it is more suited to support “sufficiency of the key mediator,” while its mechanistic necessity and exclusivity remain to be further tested (e.g., via pathway activation, genetic models, or introducing pathway intervention within the FMT framework).

### 4.3 Level B studies: Functional evidence (ABX-only or FMT-only; defined limitations)

#### 4.3.1 Tripterygium hypoglaucum extract (THH; ABX-only)

Design Type (ABX-only): A low-biomass background was established using an antibiotic cocktail in drinking water for 4 weeks to observe THH efficacy under microbiota-depleted conditions (Zheng et al., 2023) Results showed that the ameliorative effects of THH on joint swelling, inflammatory response, and bile acid metabolism abnormalities were significantly weakened or abolished after microbiota depletion (Zheng et al., 2023).

Strongest Supportable Conclusion: The anti-RA-like effect of THH possesses significant microbiota dependence (providing a necessity signal) (Zheng et al., 2023).

Limitations: Lacks FMT to verify therapeutic transferability; also missing bile acid supplementation/receptor blocking (rescue/blocking) experiments, preventing the convergence of the causal chain to “specific bile acid—specific receptor/pathway” as the key mediator.

#### 4.3.2 Fuzi (aconitum carmichaelii; FMT-only)

Design Type (FMT-only): Donor fecal microbiota was obtained from a cold-damp RA model group and a Fuzi treatment group for recipient transplantation (every 2 days until the end of the experiment), with Cold-Transpl. and Fuzi-Transpl. controls established (Liu J. et al., 2025). The microbiota of Fuzi-Transpl. recipients converged toward their donors and exhibited consistent improvement in arthritis phenotypes. Additionally, bile acids like TCA and THDCA were higher post-FMT and showed anti-inflammatory activity *in vitro* Liu J. et al., 2025).

Strongest Supportable Conclusion: The Fuzi-induced microbial state possesses therapeutic transferability; bile acids (e.g., TCA/THDCA) are high-priority candidate mediators (Liu J. et al., 2025).

Limitations: Lacks an ABX low-biomass background, making it difficult to prove microbial “necessity” and assess transfer efficiency; also missing rescue/blocking steps like bile acid supplementation or receptor (e.g., TGR5) blocking, insufficient to form a strict closed loop.

#### 4.3.3 Jingfang granules (JFG; ABX-only)

Design Type (ABX-only): An antibiotic cocktail was used for 7 days to deplete microbiota prior to JFG administration. Compared to the non-depleted JFG group, the ABX + JFG group showed significantly diminished improvements in paw swelling, synovial pathology scores, and inflammatory cytokine reduction (Wang X. et al., 2025; Wang G. et al., 2025).

Strongest Supportable Conclusion: JFG efficacy has a clear microbiota dependence (providing a necessity signal) (Wang X. et al., 2025).

Limitations: No FMT or metabolite rescue/blocking was performed, making it difficult to advance from necessity to transferability and key mediator localization. Furthermore, ABX itself may introduce non-specific immunological effects, requiring stricter controls and depletion verification metrics to reduce confounding.

### 4.4 Summary

Synthesizing the evidence from Level A/A+ and Level B studies, the current landscape can be robustly summarized as follows:

1. Microbiome dependence shows a consistent trend in various studies, but the reliability of inference still depends on the quality of missing verification. The separate antibiotic research of THH and JFG shows that their efficacy has decreased significantly after the microbiota is removed, which shows that their anti-rheumatic effect depends on the microbiota and gives a necessity signal (Zheng et al., 2023; Wang X. et al., 2025). But, due to the differences in ABX exhaustion intensity and absolute load verification, coupled with the non-specific immune interference that ABX may bring, we still need more standardized quantitative indicators and control designs to make these conclusions more reliable.
2. Transferability evidence supports that microbial changes are not merely epiphenomenal. FMT experiments with ASPS and FSN under ABX backgrounds suggest that effective donor microbiota can transfer partial therapeutic phenotypes to recipients; the FMT-only results for Fuzi also support a degree of phenotypic transferability (Liu et al., 2023; Liu J. et al., 2025; Wen et al., 2025).
3. Key mediators cluster in modules, with the “*in vivo* mediator rescue” evidence for ASPS being particularly compelling. Level A studies primarily point to two candidate modules: (i) the Butyrate/SCFAs—Barrier—Inflammation Axis (EMS, FSN), and (ii) the GGC—NLRP3 Inflammasome Axis (ASPS). The ASPS study provided high-strength support for the sufficiency of the key mediator by replicating joint protection and significantly downregulating caspase-1/IL-1β (>80%) via exogenous GGC supplementation (100 mg/kg/day, oral, starting from primary immunization) (Liu et al., 2023; Wen et al., 2025; Xu et al., 2025).
4. A+ evidence is currently provided solely by FSN, with blocking primarily limited to *in vitro* pathway intervention. FSN achieved mechanistic module convergence via HDAC/NF-κB inhibitors (TSA/PDTC) on top of an ABX + FMT + Butyrate rescue closed loop (Wen et al., 2025). However, this evidence leans more toward “mechanistic specificity enhancement.” It still requires the introduction of pathway intervention *in vivo* and within the microbial causal framework to strengthen the necessity inference and test the relative contribution of butyrate to the formula’s holistic effect (Wen et al., 2025).

## 5 Mechanistic module integration: pharmacological chains of TCM/natural products against RA mediated by gut microbiota-derived metabolites

A significant challenge in synthesizing the current evidence lies in the methodological heterogeneity across the included studies. Variations exist in animal models (CIA vs. AIA vs. CFA), intervention forms (complex formulas vs. metabolites), sampling sites (feces vs. cecal contents vs. serum), and detection platforms (16S rRNA vs. metagenomics). For instance, SCFA concentrations in cecal contents (site of production) may not linearly correlate with fecal excretion or serum exposure, and the immune baseline of CIA mice differs from that of AIA rats. To mitigate the noise introduced by these variables and avoid invalid direct comparisons of absolute abundance, we adopted a “Functional Module” approach. Instead of focusing on single bacterial species which may vary by host species or facility, we prioritized functional convergence—that is, whether different interventions in different models converge on the same functional output.

Synthesizing the current body of research through this lens, the evidence for microecology-mediated efficacy primarily converges into three distinct modules: (1) The Short-Chain Fatty Acid (SCFA) Module; (2) The Bile Acid Signaling and Tryptophan-Indole Metabolism Module (high-priority candidates, yet lacking closed-loop anchors); and (3) The Gut Barrier—Systemic Inflammation Axis, serving as a common convergence endpoint across modules. The following sections are developed according to the logic of “A-level anchor point, B-level support, C-level consistency, and unsolved problems”.

**FIGURE 4:**
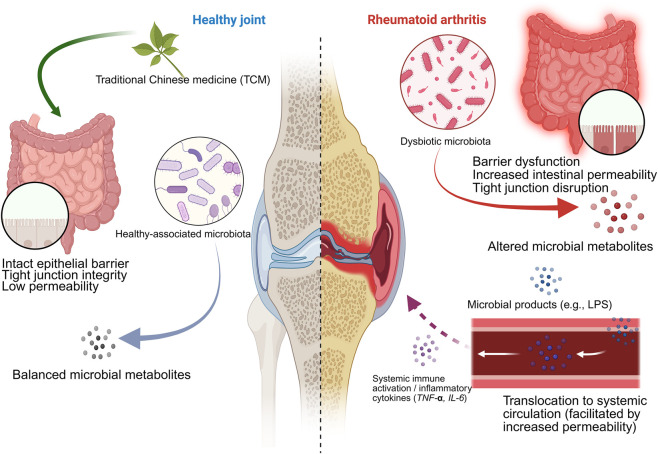
Left (Health/Steady State): The intestinal microbiota is in a health-related state, and the microbial metabolites are relatively balanced. The intestinal epithelial barrier is intact, the close connection function is good, and the intestinal permeability is low, which together maintain the immune homeosty of the whole body and joint health. Traditional Chinese medicine intervention may alleviate inflammation and joint lesions by regulating the composition and function of the microbiota, enhancing the integrity of the barrier, and reducing the transport of microbial products. To (rheumatoid arthritis/flora disorder): flora disorder is usually accompanied by impaired intestinal barrier function, broken close connection, and increased intestinal permeability. Microbial metabolites will change, and microbial products (such as lipopolysaccharide, LPS) are more likely to pass through the intestinal barrier into the systemic circulation. This triggers a systemic immune response and increases inflammatory factors (such as TNF-α, IL-6), thus promoting the progression of arthritis and rheumatoid arthritis.

Taken together, these functional modules collectively support the core mechanism that a healthy intestinal microbiota maintains immune and joint homeostasis, whereas dysbiosis impairs the intestinal barrier, triggers systemic inflammation, and promotes the progression of rheumatoid arthritis, in which traditional Chinese medicine can exert intervention effects by modulating the microbiota and intestinal barrier. The detailed mechanism and related evidence are illustrated in Figure 4.

### 5.1 The SCFAsmodule: a butyrate-centric microecology-mediated anti-inflammatory chain

#### 5.1.1 SCFAsand RA: pharmacological significance from microbial fermentation to immune balance

SCFAs, mainly acetic acid, propionic acid and butyric acid, are the key metabolites produced by intestinal microorganisms to ferment dietary fiber (Louis and Flint, 2017). They can not only serve as representative readings of the “functional output” of microorganisms, but also may act as potential remote immune regulators in the “intestinal-articular axis” by affecting the energy metabolism and barrier balance of the intestinal epithelium, and regulating the activation threshold and cytokine network of immune cells (Kasubuchi et al., 2015). In RA research, SCFAs are frequently posited as candidate functional mediators bridging “microbial alterations” and “systemic inflammation/joint damage phenotypes,” with butyrate repeatedly emerging as a priority target for verification due to its consistent association with anti-inflammatory profiles (He et al., 2022; Wang G. et al., 2025).

#### 5.1.2 Integration of evidence: butyrate as a “rescuable” priority candidate mediator

Level A/A + Anchor Evidence: The EMS and FSN studies in CIA mice provide the strongest “Microbiota—Metabolite—Phenotype” closed-loop evidence to date. Both utilized antibiotic depletion (ABX) to diminish the microecological background, verified therapeutic transferability via fecal microbiota transplantation (FMT), and further achieved partial simulation or restoration of therapeutic phenotypes through butyrate/sodium butyrate supplementation (rescue) (Wen et al., 2025; Xu et al., 2025). This supports a testable chain: “Microbial Alteration → Butyrate Restoration → Inflammation/Barrier Outcome Improvement.” It is crucial to emphasize that current closed loops more closely resemble clues of sufficiency for butyrate (supplementation brings improvement); whether butyrate constitutes a necessary mediator for efficacy still lacks blocking verification.

Level B Support: In the CFA-induced RA rat experiment, the study found that after antibiotic (ABX) treatment, the improvement effect of JFG on foot swelling, synovial lesions and inflammatory factors was significantly weakened. At the same time, the change in the level of SCFAsis consistent with the trend of efficacy, suggesting that it may depend on intestinal microecology (Wang X. et al., 2025). However, due to the lack of closed-loop experiments such as fecal bacteria transplantation (FMT) or metabolite supplementation/antagonism, these evidences are not enough to confirm that SCFAs (or butyric acid) is the key medium, so the more conservative interpretation is: “the possibility of SCFAsparticipation increases”.

Level C Consistency Supplement: Many C-level studies using different models (AIA, CIA, AA) have found that SCFAs or related functional indicators can be “recovered/reversed” with the reduction of inflammation and pathological improvement. Specifically, the study of WTD shows that SCFA-related metabolic abnormalities will be reversed during the treatment process. At the same time, TNF-α/IL-6 decreases and the barrier function is also improved (Cheng et al., 2022); In CIA rats, SXD increased SCFAs (especially butyric acid), while improving intestinal barrier indicators and immune balance (Sun et al., 2025); In AA rats, Korean white surgery can increase acetic acid/propionic acid, and butyric acid also tends to recover, which corresponds to cytokine reduction and histosology improvement (Pang et al., 2021). In CIA rats, the report shows that TIN leads to the upregulation of butyric acid and acetic acid, which is consistent with the upregulation of ZO-1/tight link protein and the downregulation of pro-inflammatory cytokines (Liu C. et al., 2025); Macrogenome analysis in the ESP study showed that the level of fecal butyric acid in treated CIA mice increased, and arthritis and cartilage damage were also improved (Ma et al., 2024); Research shows that the serum butyric acid level of CIA rats increased after taking CBD, and at the same time showed phenotypic improvement (Geng et al., 2025); In CIA rats, CTSs have been reported to correct the abnormal SCFAsspectrum and relieve arthritis (Guo et al., 2019). Additionally, a longitudinal metagenomic study of HQT combined with MTX in humans suggested functional pathway shifts during treatment, including cues related to SCFAsproduction, providing supplementary consistency at the clinical level (Mei et al., 2021), though this remains correlational. Notably, Level C studies vary in SCFAssampling sites (feces/cecal content/serum) and detection platforms; thus, their value lies primarily in elevating “candidate priority” rather than supporting causal conclusions.

#### 5.1.3 Summary box

Conclusion: In the current corpus of studies, SCFAs (particularly butyrate) are among the few candidate metabolic mediators that have entered the “ABX + FMT + Metabolite Supplementation” closed-loop verification framework, thereby commanding a high priority for actionable verification.

Evidence Boundary: While Level A studies have provided closed-loop clues of “ABX + FMT + Butyrate Rescue,” most results support suggestions of sufficiency for butyrate rather than proof of necessity. Level C consistency evidence serves only for candidate prioritization and cannot support causal inference.

##### 5.1.3.1 Priority experiments

1. Incorporate SCFAssignaling axis blockade/separation (receptor antagonism or downstream pathway inhibition) within the ABX + FMT framework to test necessity and estimate effect contribution.
2. Standardize SCFAssampling sites and quantitative platforms (reporting feces/cecum/serum separately) and supplement with blood/tissue exposure data to avoid the implicit leap that “fecal content ≈ systemic exposure.”
3. Use longitudinal time sampling (early/mid-term/late stage) to see whether the changes of SCFAs preced inflammation remission, so as to reduce the misunderstanding of causal inversion.

### 5.2 The bile acid/tryptophan module: candidate molecular clustering and evidence boundaries

#### 5.2.1 Immunopharmacological significance of bile acid and tryptophan metabolism

In addition to SCFAs, bile acids and metabolites related to tryptophan are also important functional products of microorganisms’ immune regulation of the host. (Lavelle and Sokol, 2020; Wang et al., 2023). Intestinal microorganisms can change the composition of bile acids through debinding and dehydroxylation, thus affecting their signaling characteristics and immune function in the host (Joyce and Gahan, 2017). Tryptophan metabolism may influence mucosal immune homeostasis and inflammation amplification through multiple branch pathways. In the context of RA, alterations in these metabolic pathways frequently parallel systemic inflammatory burden, barrier functional status, and local joint immune microenvironments, making them candidate mechanistic modules bridging dysbiosis and inflammatory phenotypes (Guo et al., 2025).

#### 5.2.2 Integration of evidence: bile acids/tryptophan as “priority candidates” with insufficient closed-loop localization

Based on recurrence frequency and Level B causal hints, this section focuses on the Bile Acid Signaling and Tryptophan-Indole Metabolism chains; other metabolic pathways are treated as background reprogramming phenomena rather than bases for key mediator inference.

Level A Anchor Evidence: Unlike the SCFAsmodule, the Bile Acid/Tryptophan module (and broader “comprehensive metabolic reprogramming”) lacks Level A closed-loop anchors that converge the causal chain to specific bile acid or indole metabolite molecules. Consequently, this module is better defined as a “collection of priority candidate pathways” supported jointly by Level B and C evidence, useful for generating testable hypotheses and determining verification priorities, but not to be articulated as established causal mechanisms.

Level B Support: The THH study in CFA arthritis mice showed that intervention corrected bile acid profile abnormalities and improved inflammation, but these effects vanished under ABX pseudo-germ-free conditions, placing bile acid metabolic shifts within a microbiota-dependent chain (Zheng et al., 2023). The Fuzi study in a cold-damp CIA model demonstrated that donor FMT transferred efficacy, accompanied by elevated bile acids like TCA and THDCA and changes in bile acid signaling-related pathway readouts (e.g., TGR5-cAMP-PKA axis and NLRP3-related readings) (Liu J. et al., 2025). This suggests “Bile Acid—Receptor/Signal—Inflammation Pathway” as a testable candidate chain, yet it lacks ABX necessity verification and closed-loop steps like bile acid supplementation/antagonism (Liu J. et al., 2025). Overall, Level B evidence supports bile acids as high-priority candidate mediators but falls short of converging the chain to a definitive “Specific Bile Acid Subtype—Key Receptor—Key Phenotype” causality.

Level C Consistency Supplement: Multiple Level C studies indicate broad-spectrum metabolic dysregulation in RA models, with treatment driving regression toward a healthy state across multiple pathways, paralleling microbiota structural improvement and inflammation reduction. Specifically: WTD suggested regression of bile acid and tryptophan-related metabolic abnormalities (Liu J. et al., 2025); YBTC in AIA rats indicated reversal of differential metabolites related to amino acid, bile acid, and fatty acid pathways, associated with microbiota-cytokine correlations (Shi et al., 2022); FPTF in CIA rats suggested partial reversal of tryptophan and lipid metabolism abnormalities (Qiu et al., 2025); DSD in a “Cold Stimulation + CIA” model used ^1H NMR to show regression of taurine and primary bile acid biosynthesis pathways (He et al., 2023); CBN research indicated metabolic profile remodeling paralleling anti-inflammatory/antioxidant regulation (Chen S. et al., 2023); ELP in CIA rats linked lipid inflammatory mediator metabolism (e.g., arachidonic acid) changes to joint protection (Li Y. et al., 2022); ESP in CIA mice aligned taurine/hypotaurine and arachidonic acid metabolism shifts with efficacy (Ma et al., 2024); WJT in CIA rats identified molecules like serotonin and glutathione disulfide as relevant to efficacy (Li Z. D. et al., 2022); and DBZP in CIA mice indicated reversal of multiple metabolites (including amino acid derivatives and sex hormone-related metabolites) paralleling immune pathway changes (Dong et al., 2024). Additionally, TFP and DZGP in CIA models reported serum metabolic profile alterations involving immune pathways (Li Z. et al., 2022; Zhao et al., 2024). A metabolite study of WTD (SM/PS) offered a pharmacokinetic perspective, suggesting polysaccharides influence the absorption and exposure of small molecules by altering microbiota, supplementing the “Microbiota and Pharmacokinetics and Efficacy” interaction path (Yang et al., 2024) The longitudinal macrogenome data from human HQT combined with MTX shows that the functional pathway has changed, providing some clues for the “reshaping of microecological metabolic potential” at the clinical level (Mei et al., 2021). It must be emphasized that C-level evidence is mainly used to prioritize candidate molecules and pathways, but is not enough to support causal assertions about specific bile acid/indole metabolites or receptor axes.

#### 5.2.3 Summary box

Conclusion: Bile acid and tryptophan-related metabolites (along with their synergistic reprogramming with lipid and redox pathways) constitute a “priority candidate mechanism module” repeatedly pointed to by multiple studies, potentially linking microbial changes to inflammatory pathway reprogramming.

Evidence Boundary: The current evidence is mainly grade B (hints of microbiome dependence or transferability) and C (consistency of metabolic trait regression). It lacks Level A closed loops converging the causal chain to “Specific Bile Acid/Indole Metabolite—Receptor—Phenotype,” precluding the designation of any single molecule or receptor axis as an established key mediator.

##### 5.2.3.1 Priority experiments

1. Establish a “High-Consistency Candidate List” based on molecules/pathways recurring across studies and prioritize targeted quantification (targeted LC–MS/MS) to achieve molecular-level convergence.
2. Incorporate candidate bile acid/indole metabolite supplementation or receptor/pathway antagonism into the ABX + FMT foundation to form the shortest closed loop of “Key Molecule—Key Receptor/Pathway—Key Phenotype.”
3. Standardize reporting of metabolomics platforms, sample sources, and threshold strategies, and conduct sensitivity analyses/stratified interpretations for items with inconsistent directionality to enhance reproducibility and cross-study comparability.

### 5.3 The gut barrier—systemic inflammation axis: a common convergence endpoint and mechanistic “landing point” across modules

#### 5.3.1 Module positioning: why it serves as a common endpoint

For the functional outputs of different metabolic modules to influence synovial inflammation and structural damage, they likely traverse a common conduit: “Gut Barrier Permeability Alteration—Systemic Inflammation Amplification—Peripheral Immune Activation.” (Julio-Pieper and Bravo, 2016; Liu et al., 2018). Barrier damage will allow microbial-related molecules and metabolites to enter the blood, thus changing the activation threshold of peripheral immune cells and exacerbating the inflammatory response (Chen F. et al., 2023; Di Vincenzo et al., 2024). So, positioning the intestinal barrier-systemic inflammation axis as the common confluence point of each module helps to explain the phenotypic convergence of different metabolic pathways, and also provides a framework for setting comparable common result indicators in each study. (Julio-Pieper and Bravo, 2016). In this review, “Gut Barrier” refers primarily to intestinal epithelial mechanical barriers and permeability (tight junction protein expression and functional permeability tests), supplemented by endotoxin load/microbial molecule translocation as indirect evidence of barrier breach; “Systemic Inflammation” focuses on pro-inflammatory cytokine profiles and key inflammatory pathway readouts (Di Vincenzo et al., 2024).

#### 5.3.2 Integration of evidence: evidence hierarchy for the barrier—inflammation axis as a “common landing point”

Level A Anchor Evidence: The closed-loop studies of EMS and FSN not only support butyrate as a candidate mediator but also jointly present the phenomenon of “metabolite changes accompanying barrier and systemic inflammation readout improvements,” thereby positioning the Gut Barrier—Systemic Inflammation Axis as a critical landing point for microecology-mediated effects (Wen et al., 2025; Xu et al., 2025). ASPS, through ABX + FMT combined with GGC rescue, converged efficacy to the association between specific metabolic mediators and inflammation amplification pathway readouts (e.g., NLRP3 inflammasome metrics), providing anchor evidence for “inflammation-end mechanisms” (Liu et al., 2023). It is noteworthy that even within the Level A framework, the position of barrier improvement in the causal chain (upstream driver vs. downstream concomitant) requires further clarification through temporal and blocking designs.

Level B Support: In the THH study, the simultaneous disappearance of efficacy and systemic inflammation-related improvements under ABX pseudo-germ-free conditions suggests that barrier/inflammation endpoints possess microbiota dependence (Zheng et al., 2023). The FMT transfer of Fuzi accompanied by changes in bile acid signaling axis readouts and NLRP3-related metrics suggests that alterations in “Metabolite—Inflammation Pathways” may connect to this convergence endpoint, though ABX necessity verification and closed-loop supplementation/antagonism at the molecular level are still lacking (Liu J. et al., 2025).

Level C Consistency Supplement: In Level C studies, the reduction of pro-inflammatory cytokines, alleviation of histological damage, or mitigation of bone destruction are nearly ubiquitous convergent phenotypes across interventions, supporting the stability of “systemic inflammation improvement” as a common endpoint. Crucially, some studies provided direct barrier or permeability-related readouts: WTD reported enhanced barrier function accompanying TNF-α/IL-6 reduction (Cheng et al., 2022); SXD reported ZO-1/occludin upregulation and improvement in permeability markers like DAO, ET, D-LA, and Zonulin, alongside restored Th17/Treg balance (Sun et al., 2025); TIN and ESP also reported barrier protection paralleling inflammation reduction (Ma et al., 2024; Liu C. et al., 2025). Other Level C studies, while not necessarily measuring barrier function directly, observed systemic inflammation and pathological improvements against a backdrop of “microbiota structural change/metabolic profile remodeling,” including YBTC, WJT, FPTF, ELP, DBZP, TFP, DSD, CBN, Atractylodes koreana, CTSs, and DZGP (Guo et al., 2019; Pang et al., 2021; Li Y. et al., 2022; Li Z. et al., 2022; Li Z. D. et al., 2022; Shi et al., 2022; Chen S. et al., 2023; He et al., 2023; Dong et al., 2024; Zhao et al., 2024; Qiu et al., 2025) Furthermore, the longitudinal HQT + MTX study in humans suggested clinical improvement accompanied by microecological functional pathway changes, providing consistency clues for “synchronous plasticity of systemic inflammatory status and microecological function” (Mei et al., 2021). It must be emphasized that Level C evidence mainly supports the “high-frequency occurrence of common endpoints”, but is not enough to prove the necessity or direction of barrier repair in the causal chain.

#### 5.3.3 Summary box

Conclusion: Intestinal barrier - systemic inflammation axis is common in a variety of interventions and models, so it is the most suitable “convergence endpoint/mechanism landing point” for different metabolic modules to achieve remote immune regulation.

Evidence Boundary: Although A/B-level studies show that this endpoint depends on the microbiota and is related to changes in metabolites, the existing evidence is not enough to determine the direction of barrier repair in the causal chain (upstream drive or downstream accompaniment). The C-level results only support the consistency of this common endpoint, not its necessity.

##### 5.3.3.1 Priority experiments

1. Through longitudinal research and intermediary analysis, the time relationship of “barrier improvement - inflammation reduction” was ligid out, and verified through intervention in the critical time window.
2. Establish the most basic barrier assessment combination: do at least one functional permeability test (such as FITC-dextran), add a closely connected protein test (such as ZO-1/occludin/claudin), and add an endotoxin/inflammatory index determination, which can enhance the explanatory and comparability.
3. In ABX/FMT research, it is necessary to strictly control the mixed factors (removement effect, cage/batch effect, non-specific immune effect of antibiotics), and report the verification index of flora colonization/transfer, so as to avoid misting the methodological pseudo-effect as a mechanism effect.

## 6 Discussion

### 6.1 Principal findings: Evidence stratification and core conclusions

Based on systematic database retrieval, this review establishes an evidence base guided by the “correlation to causality” grading framework, which is used to integrate the stratification and mechanism of research on traditional Chinese medicine/natural products, rheumatoid arthritis (RA) and intestinal microorganisms from 2015 to 2025. Among the 25 studies included, the distribution showed pyramidal scarcity: 2 A-level, 1 A + -level, 3 B-level and 19 C-level studies; in addition, there is a clinical longitudinal study that is only used as evidence of correlation to support consistency, not causal inference. Overall, data across various animal models and limited clinical observations have repeatedly shown a trend: interventions are usually accompanied by changes in the composition and functional output of intestinal microorganisms (metabolite spectrum), and occur synchronously with the improvement of arthritis phenotype. However, evidence robust enough to support a testable causal chain of “Microbiota—Metabolite—Immune Inflammation—RA Phenotype” is currently concentrated within a handful of Level A/A+ studies. The majority of research remains tethered to Level C or partial Level B status, necessitating that mechanistic claims be strictly confined within these evidentiary boundaries.

At the mechanistic module level, the Short-Chain Fatty Acid (SCFA) chain—particularly butyrate—possesses relatively higher testability. A minority of Level A/A+ studies have successfully converged microbial changes to the metabolite level via the path of “ABX depletion effect + FMT transferability + Butyrate rescue,” suggesting that butyrate is likely one of the priority candidate functional mediators (Wen et al., 2025; Xu et al., 2025). In contrast, bile acids/tryptophan and broader metabolic pathway reprogramming fit the definition of “priority candidate modules”: current evidence indicates that bile acid profiles, indole-related tryptophan metabolites, or lipid inflammatory mediator pathways may regress toward homeostasis concurrently with inflammation relief. Yet, these pathways lack the closed-loop verification required to converge the chain to a “Specific Molecule—Receptor/Pathway—Phenotype” sequence, making them more suitable as a candidate pool for the next phase of causal verification (Zheng et al., 2023; Liu C. et al., 2025). Furthermore, the Gut Barrier—Systemic Inflammation Axis repeatedly surfaces as a common outcome across multiple studies, implying it may serve as a critical “mechanistic locus” where the effects of distinct metabolic modules converge. Nevertheless, its directionality (upstream driver vs. downstream concomitant) and necessity within the causal chain await clarification through longitudinal and blocking studies.

### 6.2 Candidate prioritization: recurring signals from correlational evidence

Although Level C studies constitute the majority (76%) of the included literature and cannot independently establish causality, they provide a rich repository of “recurring signals” that serve as a roadmap for future hypothesis testing. By systematically synthesizing findings across diverse models (CIA, AIA) and interventions (formulas, metabolites), we identified a “High-Priority Verification List” (Table 4). Unlike the uniform “depletion-restoration” pattern often assumed, the observed trends reveal a complex landscape of ecological remodeling, where therapeutic success is linked to the rebalancing of specific high-frequency taxa rather than a unidirectional shift.

**TABLE 4:** Priority list for future validation: High-Frequency candidates derived from level C evidence. Note: This list summarizes candidates that appeared in ≥3 independent studies with consistent regulatory trends.

Category	Priority candidate	Observed trend (RA model → treatment)	Proposed mechanism/Hypothesis	Representative studies (Ref.)
Microbial taxa	Prevotella spp.	Predominantly suppressed (mixed in some models)	Reduction of Th17 activation; Lowering pro-inflammatory LPS load	Suppressed: (Guo et al., 2019; Cheng et al., 2022; Shi et al., 2022; Yang et al., 2024; Wen et al., 2025) Enriched: (Li et al., 2022b; Sun et al., 2025)
	*Lactobacillus*	Variable/predominantly decreased	Immune modulation; Ecological niche re-adjustment	Decreased: (Li et al., 2022b; He et al., 2023; Dong et al., 2024; Liu et al., 2025a; Sun et al., 2025; Wang et al., 2025b) Increased: (Cheng et al., 2022; Li et al., 2022a; Chen et al., 2023b; Qiu et al., 2025)
	Akkermansia	Variable/context-dependent	Mucin regulation; Barrier reinforcement vs. Overgrowth control	Decreased: (Pang et al., 2021; Cheng et al., 2022; Yang et al., 2024; Sun et al., 2025) Increased: (Li et al., 2024; Qiu et al., 2025)
	*Bacteroides*	Bidirectional regulation	SCFA production vs. Opportunistic pathogenicity	Enriched: (Li et al., 2022b; He et al., 2023; Dong et al., 2024; Liu et al., 2025b; Sun et al., 2025) Suppressed: (Cheng et al., 2022; Liu et al., 2023; Yang et al., 2024; Xu et al., 2025)
	Butyrate producers (butyricicoccus, faecalibacterium)	Mixed/restored in specific subsets	SCFA production; HDAC inhibition	Restored/Increased: (Li et al., 2022b; Qiu et al., 2025; Sun et al., 2025) Decreased: (Li et al., 2022a; Xu et al., 2025)
Metabolites	SCFAs (short-chain fatty acids)	Decreased → increased	AMPK activation; Tight junction repair	Liu et al. (2025a), Sun et al. (2025), Wang et al. (2025b)
	Bile acids	Dysregulated → normalized	TGR5/FXR signaling; NLRP3 inhibition	Cheng et al. (2022), Zheng et al. (2023), Liu et al. (2025b)
	Tryptophan metabolites	Decreased → increased	AhR activation; Mucosal homeostasis	Cheng et al. (2022), Qiu et al. (2025)
Pathways	Gut barrier function	Impaired → repaired	Upregulation of ZO-1/Occludin	Cheng et al. (2022), Liu et al. (2025a), Sun et al. (2025)

#### 6.2.1 Microbial taxa

The “Paradox” of Beneficial Taxa: Contrary to the common expectation that beneficial bacteria are universally restored, our analysis reveals a dual-directional regulation. While some interventions restored Butyricicoccus and Faecalibacterium [e.g. (Li Z. et al., 2022; Sun et al., 2025)], others significantly reduced the abundance of *Lactobacillus* and Akkermansia [e.g. (Cheng et al., 2022; Wang X. et al., 2025; Sun et al., 2025)], suggesting that effective TCM treatment may involve reducing the overgrowth of specific commensals in inflammatory contexts or reshaping the competitive niche.

Pathobiont Suppression: The suppression of potential pathobionts remains a strong signal. Genera such as Prevotella and Desulfovibrio were frequently downregulated alongside inflammation relief (Table 5), although specific strains exhibited variable responses depending on the intervention type.

**TABLE 5:** Summary of significant alterations in gut microbiota genera induced by botanical drugs and plant metabolites.

Intervention	UP microbiota	Down microbiota	References
Fengshining Decoction (FSN)	Butyrivibrio Faecalicatena Lacrimispora	Alistipes *Proteus* Muribaculum Prevotella	Wen et al. (2025)
Er Miao San (EMS)	Bacteroidota	*Bacteroides* Faecalibacterium	Xu et al. (2025)
*Acanthopanax senticosus* polysaccharides (ASPS)	Ruminococcus Blautia Colidextribacter GCA-900066575 Acetatifactor	*Bacteroides*	Liu et al. (2023)
*Tripterygium hypoglaucum* extract (THH)	Not reported	Not reported	Zheng et al. (2023)
*Aconitum carmichaelii Debx* extract (Fuzi)	Lachnospiraceae Ruminococcaceae Peptostreptococcaceae Erysipelotrichaceae Alloprevotella NK4A214-group *Bacteroides* UCG-005 Phascolarctobacterium	Eggerthellaceae Enterobacteriaceae Turicibacter	Liu et al. (2025b)
Jingfang Granules (JFG)	• Firmicutes • norank_f__Muribaculaceae unclassified_f__Lachnospiraceae	• Bacteroidota • *Lactobacillus* norank_f__norank_o__Clostridia_UCG-014	Wang et al. (2025b)
Wu-tou decoction (WTD)	*Lactobacillus* Oscillospira	*Bacteroides* Prevotella Akkermansia *Enterococcus* Dorea Jeotgalicoccus	Cheng et al. (2022)
Wu-tou Decoction (WTD); fractions (SM vs. PS)	Bacteroidetes Tenericutes Oscillospira Bifidobacterium Bacteroides	Firmicutes Unspecified Clostridiaceae Prevotella Akkermansia The inhibitory effect unique to the WTD group Dorea Turicibacter *Streptococcus* Adlercreutzia	Yang et al. (2024)
Yaobitong capsule (YBTC)	Eubacterium Bilophila Lachnoclostridium Oscillibacter Ruminiclostridium_9 Intestinimonas Ruminococcus_1 Ruminiclostridium	Muribaculaceae_unclassified Prevotella Turicibacter	Shi et al. (2022)
Wantong jingu tablet (WJT)	Bacteroidetes Tenericutes Deferribacteres	*Vibrio* Macrococcus Vagococcus	Li et al. (2022c)
Siweixizangmaoru decoction (SXD)	Bacteroidetes *Bacteroides* Butyricicoccus Oscillibacter Lachnospiraceae_NK4A136_group Lachnospira Prevotellaceae_UCG-001 Prevotella	Firmicutes Desulfovibrio *Lactobacillus* Akkermansia *Helicobacter* Dubosiella Allobaculum	Sun et al. (2025)
*Flemingia philippinensis* total flavonoids (FPTF)	*Lactobacillus* Akkermansia Butyricicoccus Colidextribacter Roseburia Lachnospiraceae_NK4A136_group	Prevotellaceae_NK3B31_group Ruminococcus Alloprevotella Gordonibacter Prevotellaceae_UCG-001 Parasutterella	Qiu et al. (2025)
Ershiwuwei Lvxue Pill (ELP)	*Lactobacillus* Lachnospiraceae_unclassified Prevotellaceae_NK3B31_group Quinella	Collinsella Dorea [Eubacterium]_ventriosum_group Anaerostipes Coprococcus_1 Ruminiclostridium_5 Ruminococcus_1 Family_XIII_UCG-001 Butyricicoccus Erysipelotrichaceae_UCG-003 Lachnoclostridium Faecalibacterium Lachnospiraceae_UCG-010 Roseburia Rs-E47_termite_group_norank *Treponema*_2	Li et al. (2022a)
Dianbaizhu (*G. leucocarpa*) polysaccharides (DBZP)	*Bacteroides* Alistipes Mucispirillum norank_f__norank_o__Rhodospirillales Colidextribacter Tuzzerella Parvibacter Muribaculum	*Lactobacillus* Enterorhabdus Candidatus_Saccharimonas UCG-005 Candidatus_Arthromitus Anaerofustis	Dong et al. (2024)
Berberine (BBR)	Akkermansia Blautia Eubacterium • Prevotellaceae_UCG_001	Prevotella_9	Li et al. (2024)
Twenty-five Wei’er tea pill (TFP)	*Bacteroides* Eubacterium Prevotellaceae_UCG-001 Blautia Faecalibacterium	*Lactobacillus* Romboutsia Christensenellaceae_R-7_group	Li et al. (2022b)
Tinosporine (TIN)	Desulfovibrio *Helicobacter* Bifidobacterium Ruminococcus Proteobacteria	*Lactobacillus* Ruminococcus Mucispirillum *Clostridium* Firmicutes Bacteroidetes Deferribacteres	Liu et al. (2025a)
Cannabidiol (CBD)	Allobaculum_unclassified Allobaculum_fili	Prevotella_unclassified Bacteria_unclassified	Geng et al. (2025)
Ephedra sinica polysaccharide (ESP)	Dubosiella Roseburia Bifidobacterium, *Clostridium* Pseudoramibacter Faecalibaculum Parabacteroides Dubosiella newyorkensis *Clostridium* sp. 26_22 Bifidobacterium pseudolongum Parabacteroides chinchillae	Alistipes *Enterococcus* Enterorhabdus Odoribacter *Escherichia* Enterorhabdus caecimuris *Escherichia coli* *Enterococcus* faecium	Ma et al. (2024)
Danggui Sini Decoction (DSD)	Romboutsia norank_f_Eubacterium_coprostanoligenes_group Bacteroides	Ruminococcus_torques_group Lactobacillus Dubosiella	He et al. (2023)
Columbianadin (CBN)	Lachnospiraceae Rikenellaceae *Lactobacillus*	Muribaculaceae Ruminococcaceae Intestinimonas	Chen et al. (2023b)
*Atractylodes koreana* (Nakai) Kitam extract	Erysipelotrichaceae	Akkermansia Allobaculum Anaerovorax	Pang et al. (2021)
Clematis total saponins (CTSs)	f_Coriobacteriaceae_UC g_Allobaculum g_Mogibacterium g_Ruminococcus o_Clostridiales_UC f_Ruminococcaceae_UC g_Blautia g_Brachybacterium g_*Clostridium*.sensu.stricto g_Bifidobacterium g_*Clostridium*.IV g_Turicibacter g_*Staphylococcus* g_*Streptococcus*	p_Proteobacteria_UC g_Paraprevotella g_Prevotella g_Vampirovibrio g_Parasutterella g_Oscillibacter f_Porphyromonadaceae_UC f_Desulfovibrionaceae_UC g_Alloprevotella	Guo et al. (2019)
Huayu-Qiangshen-Tongbi formul + methotrexat HQT + MTX (vs. LEF + MTX)	*Clostridium* celatum Roseburia inulinivorans Turicibacter sanguinis *Pasteurella* bettyae Burkholderiales bacterium 1-1-47(Boosted at months 3 and 6)	*Clostridium* symbiosum Clostridiales bacterium 1-7-47FAA *Enterobacter* aerogenes	Mei et al. (2021)
Duzheng Pian (DZGP)	Verrucomicrobiota Cyanobacteria Bacteroidota Fusobacteriota	Firmicutes Proteobacteria Euryarchaeota	Zhao et al. (2024)

#### 6.2.2 Metabolites and pathways

Beyond SCFA modulation, Bile Acid metabolism (e.g., secondary bile acid transformation) and Lipid/Amino Acid metabolism (e.g., arachidonic acid cascade, tryptophan metabolism) appear as prominent metabolic signatures. These pathways likely bridge the gap between microbiota alterations and downstream immune targets such as the NLRP3 inflammasome and Th17/Treg balance.

We recommend that future studies prioritize these candidates for targeted quantification. Specifically, validation should focus on clarifying the context-dependent roles of Akkermansia and Lactobacillus—determining whether their reduction represents a return to homeostasis from a dysbiotic expansion or a side effect of broad-spectrum antimicrobial activity.

### 6.3 Comparison with literature: consistency, divergence, and interpretation

Taking Wu-tou decoction (WTD) as a case study, two investigations based on the adjuvant-induced arthritis (AIA) rat model delineate the “Formula—Microecology—Efficacy” structure from distinct cross-sections, forming a somewhat complementary evidence chain. Cheng et al. (Cheng et al., 2022) used 16S rRNA sequencing plus targeted metabolomics. They found that WTD improved arthritis phenotypes. At the same time, it changed the gut microbiota structure and also changed several functional outputs. These included changes of SCFAs, bile acids, and tryptophan-related metabolites. Overall, these metabolite signals moved back toward a more normal state. These changes happened together with better gut barrier-related readouts and lower pro-inflammatory cytokines (TNF-α and IL-6). The value of this study is that it does not only list “different genera”. It goes further to the metabolite profile level. From the functional output view, it supports a combined story: multiple metabolic modules are regulated in parallel, and they show a common end point at systemic inflammation measures. But we should also be careful. Their conclusion mainly comes from multi-omics association and pattern consistency. It is still not a strict causal chain. Strong causality will need later studies to test key nodes with real interventions (for example rescue or blocking).

Different from Cheng et al., who mainly talked about the parallel change of “microbiota—metabolite—phenotype”, Yang et al. (2024) split WTD into two parts. One part was small molecules (SM), and the other part was polysaccharides (PS). They compared WTD, SM, and PS for RA phenotypes and gut microbiota changes. The study showed that SM alone and PS alone could still affect the microbiota to some degree. But the full WTD formula worked better. It was better both in strength and in coverage. This result suggests that the best effect may need the combined action of different parts. It may not be fully copied by one fraction only. On the microbiota side, the overall direction was similar across the three treatments. It looked like “more possibly helpful bacteria, and less disease-related taxa”. At the phylum level, Bacteroidetes and Tenericutes increased, while Firmicutes decreased. At the genus level, Oscillospira and Bifidobacterium increased, but unclassified Clostridiaceae, Prevotella, and Akkermansia decreased. Another clear point was that the full WTD formula suppressed some genera more strongly, such as Dorea, Turicibacter, *Streptococcus*, and Adlercreutzia. This inhibition was much weaker in the SM or PS groups. This gap suggests that the “stronger and wider” microbiota change from the whole formula is not just a replaceable effect from one part. This pattern aligns with recent findings on Polyporus Umbellatus polysaccharide (PUP), which similarly demonstrated that polysaccharide interventions could remodel specific genera like Odoribacter and Alistipes while inhibiting the NF-κB pathway to alleviate joint edema (Zheng et al., 2025). It is more likely from the combined interaction between metabolites.

We should be careful about the “direction” of metabolite changes across studies. It is not always the same. The differences may come from basic non-match between studies, like sampling site (cecum vs. feces vs. serum), disease stage, test platform, and which metabolites are actually covered. Guo et al. reported that many SCFAs were abnormally high in the cecal contents of CIA rats. At the same time, Clematis total saponins (CTSs) improved arthritis phenotypes, and these unusually high SCFAs went down back toward more normal levels (Guo et al., 2019). This result suggests a clear point. At least for luminal concentration, “higher SCFAs” does not always mean better protection. It may instead show disease-related problems, such as abnormal fermentation, poor absorption or use, or wrong spatial distribution. So, when we combine results across studies, it is safer to say “correction back to homeostasis” or “remodeling of spatial distribution”, instead of using a simple “more is better” rule. Also, the sample source should be stated clearly, or we may mix readouts that cannot be compared.

### 6.4 Strengths and limitations: advantages, boundaries, and alternative explanations

An obvious advantage of this review is the introduction of evidence classification and the integration of cross-model evidence through “mechanical modules”, thus distinguishing a large number of correlation studies from a few testable closed-loop studies, reducing the risk of over-interpretation of mechanisms. At the same time, there are some restrictions that need attention. First of all, A-level/A+ closed-loop evidence is scarce, and most mechanisms are still in the “priority candidate” stage, which is not enough to identify key media. Secondly, although ABX is often used to assess microbial dependence, antibiotics will inevitably affect the host’s immunity and barrier homeostasis through non-microbial pathways. If there is no verification of clearance efficiency, sufficient control and FMT supplementation, it is very risky to attribute the effect change under ABX conditions alone to microorganisms. Third, FMT studies reported inconsistency in flora colonization verification, control settings and cage/batch control, which reduces the reviewability of transferable evidence. Fourth, there is obvious heterogeneity in metabolite research: differences in sampling sites, disease stages, detection platforms and metabolite coverage may lead to directional inconsistency. Therefore, in cross-research integration, “regression homeostasis” is more comparable than “single upward/downward adjustment” (Guo et al., 2019). Fifth, while animal models provide an operable platform for mechanistic verification, their immune triggers differ from human RA, making them more suitable for testing falsifiable hypotheses than for making direct clinical mechanistic equivalencies. Sixth, one must remain vigilant against reverse causality: inflammation relief may indirectly affect microbiota and metabolite readouts via changes in dietary intake, bile acid cycling, intestinal motility, and niche alterations. Thus, longitudinal timing and interventional designs are essential to locate key nodes. Finally, animal research and metabolomics research are susceptible to the publication of bias and selective reports, which further reduces the repeatability of cross-study.

Our SYRCLE risk of bias assessment (Figure 3) highlights a specific limitation: the widespread lack of reporting on “allocation concealment” and “blinding” (both performance and detection). In preclinical TCM research, the absence of blinding may lead to an overestimation of treatment effects. Furthermore, the predominance of “Unclear” risks suggests that while the study design might be sound, the reporting standards need significant improvement to rule out selection and detection biases. Therefore, the causal inferences drawn in this review should be interpreted with caution, acknowledging that the “efficacy” observed may be partially influenced by these methodological gaps.

Crucially, a critical assessment of the included literature reveals significant pharmacological limitations, particularly among Level C studies. Many studies failed to report the full taxonomic validity of the plant materials or the drug-extract ratio (DER), which compromises reproducibility. Furthermore, several studies lacked rigorous dose-response analyses or appropriate positive/negative controls to validate the specificity of the observed microbiome shifts. Consequently, while the “microbial shifts” are well-documented, the pharmacological specificity of these effects often remains ambiguous.

### 6.5 Implications and future directions: roadmap for inquiry

Considering the current evidence status and inadequacy of methods, the research that wants to upgrade from “related networks” to “falsible causal chains” in the future should take the route of “shortest closed loop priority” and establish minimum reporting standards for key steps. (1) Standardize Depletion Verification and Reporting:When using antibiotics (ABX), the composition, dosage, time of use and route of use of the drug should be clearly stated. The most important thing is to provide at least one verification of consumption efficiency (such as absolute quantification of fecal microbial load through 16S copy number qPCR or culture count), and record/control interfering factors, such as cage/batch effect, food intake, weight and disease severity. (2) Elevate FMT Evidence Quality: FMT research should fully report the program of donor processing and receptor preparation. Verification of bacterial colonization is necessary - do the changes in the characteristic bacterial/functional pathways or metabolites of the donor appear synchronously in the receptor? Regarding the control, the most basic is at least a carrier control; ideally, autologous FMT or heat-treated FMT should be added to enhance the causal explanation. In statistical analysis, “animals” should be the unit, and the cage effect should be considered at the same time. Recent emerging studies, such as the investigation into Aconitum pendulum polysaccharides (APPs), have explicitly recognized this limitation, stating that while microbial shifts were observed, causal verification via FMT remains essential for future validation (Meng et al., 2025). (3) Prioritize Rescue and Blocking Sequence: We suggest that quantitative convergence should be achieved for those candidate molecules (such as butyrates, specific bile acid subtypes, and typical indole metabolites) that repeatedly appear in different studies. Then, it can be verified whether it is sufficient through metabolite supplementation (rescue) under the framework of ABX + FMT, and finally test the necessity and estimate the contribution ratio through receptor/pathway blocking (*in vivo* blocking), thus forming the shortest closed-loop evidence chain of “key molecules-key receptors/pathways-key phenotypes”. (4) Two Viable Translational Paths: First of all, it is necessary to give priority to including comparative functional indicators (target metabolite quantification, barrier markers, longitudinal changes in inflammation results) in clinical and preclinical studies to enhance the testability of the description of “functional output to systemic inflammation”. Second, leveraging the “Microecology—Pharmacokinetics Interaction” framework, integrate microbial changes, key metabolite exposure (AUC/Cmax), and therapeutic outcomes into a single analytical system. This provides a quantifiable, falsifiable mechanistic explanation for the holistic efficacy of formulas and improves the operability of extrapolating animal results to the clinic (Yang et al., 2024).

### 6.6 Bridging the gap: challenges and strategies for clinical translation

Given that only one clinical study (Level C) was identified in this review, a substantial gap remains between animal findings and human application.

#### 6.6.1 Major obstacles

Interspecies Variation: The murine gut microbiome overlaps by only ∼4% with the human gene catalog (Xiao et al., 2015). Disease Complexity: Human RA involves long-term courses and complex co-medications (e.g., MTX) difficult to mimic in acute animal models. The Bioavailability Gap: In animals, gavage ensures exposure; in humans, microbial enzymes may degrade TCM metabolites before absorption, creating “non-responders (Zimmermann et al., 2019).”

#### 6.6.2 Viable translational paths: to bridge this gap, we propose integrating the following strategies

Clinical and Preclinical Alignment: Prioritize comparative functional indicators (e.g., targeted metabolite quantification, barrier markers like Zonulin/LPS) over mere taxonomic lists to enhance the testability of “functional output” in humans. Longitudinal and Humanized Models: Utilize humanized gnotobiotic mice or longitudinal clinical cohorts with dense sampling to distinguish whether microbiome shifts are the cause or consequence of remission. The “Microbiota–Pharmacokinetics (PK)” Framework: As suggested by Yang et al. (2024), future studies should integrate microbial changes, key metabolite exposure (AUC/Cmax), and therapeutic outcomes into a single analytical system. This provides a quantifiable, falsifiable mechanistic explanation for the holistic efficacy of formulas and improves the operability of extrapolating animal results to the clinic.

## 7 Conclusion

Synthesizing the evidence from 2015 to 2025, a high degree of consistency emerges between Traditional Chinese Medicine/natural product interventions for Rheumatoid Arthritis (RA) and the remodeling of gut microbiota structure and its functional output (metabolite profiles), phenomena that frequently parallel inflammation relief and improvement in joint pathology. However, evidence robust enough to support a testable causal chain of “Microbiota—Metabolite—Immune Inflammation—RA Phenotype” is currently concentrated in a sparse collection of Level A/A+ studies; the vast majority of research remains arrested at the correlational or partial functional verification stage. Consequently, mechanistic articulations must be strictly confined within these evidentiary boundaries.

Regarding the priority of testable mechanisms at this stage, the SCFAsmodule (specifically butyrate) commands a relatively higher verification priority due to the existence of closed-loop clues involving “ABX depletion effect + FMT transferability + Metabolite rescue.” Conversely, bile acids/tryptophan and multi-metabolic pathway reprogramming are better suited as a pool of candidate mechanisms, still requiring convergence to the level of key molecules and receptors/pathways through “Supplementation/Blocking” shortest closed-loop experiments. The Gut Barrier—Systemic Inflammation Axis, appearing repeatedly across multiple studies as a common endpoint across modules, represents a critical locus, yet its directionality and necessity within the causal chain await clarification through longitudinal and blocking research.

Future inquiries that build upon standardized depletion verification and FMT evidence quality, advancing along the path of “Targeted Candidate Molecule Convergence—Rescue Verification of Sufficiency—*In Vivo* Blocking Test of Necessity,” and incorporating quantifiable frameworks like “Microecology—Pharmacokinetics Interaction,” will stand a far greater chance of forging a mechanistic evidence chain that is reproducible, falsifiable, and holds genuine translational value.
